# IL6 Derived from Macrophages under Intermittent Hypoxia Exacerbates NAFLD by Promoting Ferroptosis via MARCH3‐Led Ubiquitylation of GPX4

**DOI:** 10.1002/advs.202402241

**Published:** 2024-09-04

**Authors:** Weisong Cai, Sa Wu, Xiaoping Ming, Zhen Li, Dingyu Pan, Xiuping Yang, Minlan Yang, Yufeng Yuan, Xiong Chen

**Affiliations:** ^1^ Department of Otorhinolaryngology Head and Neck Surgery Zhongnan Hospital of Wuhan University Wuhan 430071 China; ^2^ Sleep Medicine Center Zhongnan Hospital of Wuhan University Wuhan 430071 China; ^3^ Department of Gynaecology II Maternal and Child Health Hospital of Hubei Province Tongji Medical College Huazhong University of Science and Technology Wuhan 430070 China; ^4^ Department of Hepatobiliary and Pancreatic Surgery Zhongnan Hospital of Wuhan University Wuhan 430071 China; ^5^ Bariatric and Metabolic Disease Surgery Center Zhongnan Hospital of Wuhan University Wuhan 430071 China

**Keywords:** IL6, MARCH3, NAFLD, OSA, ubiquitination

## Abstract

Obstructive sleep apnea (OSA) is a common sleep disorder characterized by intermittent hypoxia (IH) and is associated with the occurrence and development of nonalcoholic fatty liver disease (NAFLD). However, the specific mechanism by which OSA induces NAFLD remains unclear. Therefore, effective interventions are lacking. This study aims to investigate the role and mechanism of ferroptosis in OSA‐related NAFLD using clinical data analyses, cell‐based molecular experiments, and animal experiments. Indicators of liver function, lipid accumulation, and ferroptosis are also examined. RNA‐seq, qPCR, western blotting, gene intervention, and E3 ligase prediction using UbiBrowser and co‐IP are used to explore the potential underlying mechanisms. The results show that ferroptosis increases in the liver tissues of patients with OSA. Chronic IH promotes NAFLD progression in mice and is alleviated by a ferroptosis inhibitor Fer‐1. The increased secretion of IL6 by macrophages can promote the expression of MARCH3 in hepatocytes under intermittent conditions, and subsequently promote the ubiquitination and degradation of GPX4 to regulate ferroptosis and lipid accumulation in hepatocytes. Hence, targeted inhibition of MARCH3 may alleviate IH‐induced ferroptosis and lipid accumulation in liver tissues and inhibit the progression of NAFLD.

## Graphical abstract

The study identifies that the upregulation of MARCH3 is a vital mechanism under intermittent hypoxia for IL6‐induced ubiquitination and degradation of glutathione peroxidase 4 (GPX4) in hepatocytes. Targeting the inhibition of MARCH3 can alleviate liver ferroptosis and lipid accumulation induced by intermittent hypoxia, thus slowing the progression of nonalcoholic fatty liver disease (NAFLD).

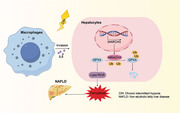

## 1 Introduction

Obstructive sleep apnea syndrome (OSA) is a common sleep disorder worldwide. It is caused by upper airway collapse and stenosis, and results in long‐term chronic hypoxia, hypercapnia, and sleep disorders.^[^ ^1^ ^]^ Many basic and clinical studies have shown that OSA is associated with multiple organ damage, such as the heart, brain, lungs, kidneys, and other organs, and endocrine system disorders, and is an independent risk factor for hypertension, coronary heart disease, diabetes, stroke, metabolic syndrome, and other diseases.^[^ ^2^ ^]^ Nonalcoholic fatty liver disease (NAFLD) is a group of clinicopathological syndromes caused by excessive fat deposition in liver cells, alcohol consumption, and other specific liver injury factors.^[^ ^3^ ^]^ NAFLD exacerbates liver‐related complications, such as hepatocellular tumors, end‐stage liver disease, cardiovascular disease, chronic kidney disease, and diabetes.^[^ ^4^ ^]^ In recent years, clinical studies have confirmed the close correlation between OSA and NAFLD. A recent meta‐analysis showed that the rates of NAFLD in patients with simple snoring and those with mild, moderate, and severe OSA were 42.86%, 63.5%, 79.4%, and 79.2%, respectively. The severity of NAFLD was positively correlated with the severity of OSA.^[^ ^5^, ^6^ ^]^ However, their mutual relationships and underlying mechanisms remain unclear, and effective prevention and intervention measures are scarce.^[^ ^7^ ^]^

Initially, the pathogenesis of NAFLD was widely accepted as the “second strike” theory. The first strike is the accumulation of triglycerides and other lipids, and the second is the accumulation caused by the release of free radicals by cytokines and inflammatory mediators, which further exacerbates abnormal lipid metabolism.^[^ ^8^ ^]^ However, with the expansion of related research, increasing evidence suggests that the theory of multiple parallel strikes, including oxidative stress, endoplasmic reticulum stress, lipid metabolism disorders, abnormal adipokine and cytokine generation, and mitochondrial dysfunction, can better explain the causes of NAFLD occurrence and development.^[^ ^9^ ^]^ However, there is no clear sequence of lipid accumulation or peroxidation induced by these factors; rather, they mutually promote each other.^[^ ^10^ ^]^ Ferroptosis is an iron‐dependent mode of programmed cell death that differs from apoptosis, necrosis, and autophagy.^[^ ^11^ ^]^ The main mechanism of ferroptosis is the generation of ROS and hydroxyl free radicals during the Fenton reaction through an iron‐dependent mechanism, which catalyzes the high expression of unsaturated fatty acids on the cell membrane to promote lipid peroxidation, thereby inducing cell death. Lipid peroxidation associated with liver cell ferroptosis plays an important role in the occurrence and development of NAFLD.^[^ ^12^, ^13^ ^]^ In our previous study, iron overload was observed in the serum of patients with OSA.^[^ ^14^ ^]^ Therefore, we speculated that ferroptosis might play a role in OSA‐related NAFLD.

NAFLD is a type of low‐grade chronic inflammation associated with obesity, insulin resistance, type 2 diabetes mellitus (T2DM), hypertension, hyperlipidemia, and metabolic syndrome. The liver is the main immune organ in the body and is rich in various innate immune cells, including macrophages and dendritic cells. Activation of these cells further coordinates multiple innate immune responses, triggering liver inflammation. Numerous experimental and clinical data indicate that macrophages, the most abundant immune cells in the liver, play a central role in the development of NAFLD and that proinflammatory macrophages determine its severity.^[^ ^15^, ^16^ ^]^ In addition, macrophages play an important role in liver ischemia‐reperfusion injury. Similarly, previous studies have indicated that intermittent hypoxia (IH) has no significant effect on lipid accumulation or peroxidation in hepatocytes.^[^ ^17^, ^18^, ^19^ ^]^ Therefore, we investigated the role of macrophages in the present study. Remarkably, we found that IH induced macrophage infiltration and increased the secretion of IL6 by macrophages in the liver, which induced lipid accumulation and promoted lipid peroxidation by inducing the ubiquitination‐mediated degradation of glutathione peroxidase 4 (GPX4) in hepatocytes. This process depends on the participation of the ubiquitin ligase MARCH3. Our study suggests that the targeted inhibition of IL6 or MARCH3 could reduce the development of OSA‐induced NAFLD, providing new ideas for the prevention and treatment of OSA‐related NAFLD.

## 2 Results

### 2.1 Ferroptosis Increased in the Liver Tissues of Patients with OSA

**Figure 1:**
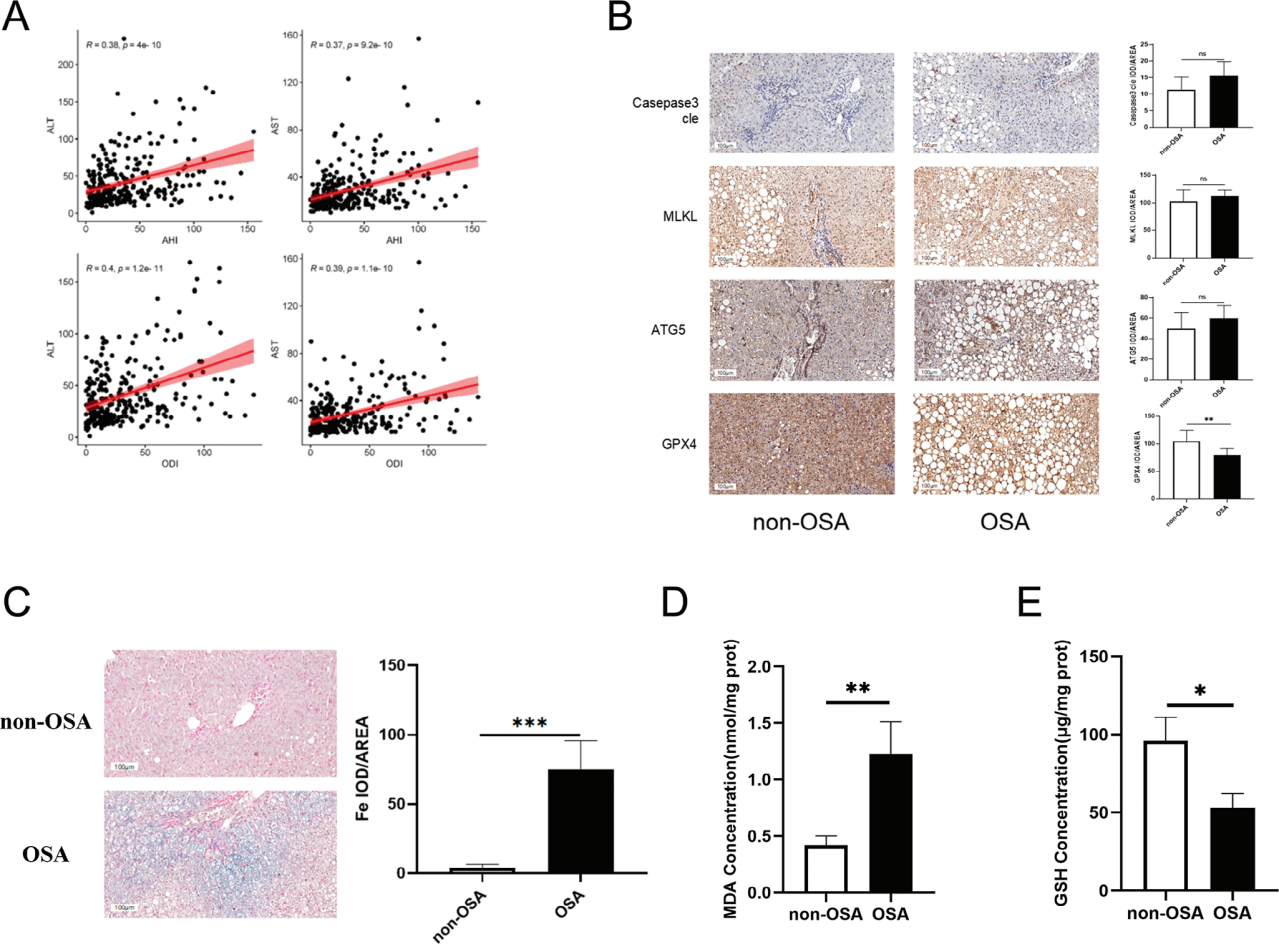
Ferroptosis increased in the liver tissues of patients with OSA. A) Correlation analyses between liver function indicators (ALT and AST) and key indicators of OSA (AHI and ODI) (*n* = 270). B) IHC analysis of key proteins expressed via different pathways of programmed death (scale bar, 100 µm). C) Prussian blue staining of iron (scale bar, 100 µm). D,E) Levels of MDA and GSH in liver tissues of patients (non‐OSA = 10, OSA = 10). All bar charts shown represent the mean ± SEM, ^*^ *p* < 0.05, ^**^ *p* < 0.01, ^***^ *p* < 0.001, ns, not significant, by *t*‐test.

We aimed to determine whether the level of ferroptosis differed in the liver tissues of patients with OSA. Correlation analyses between liver function indicators (ALT and AST) and key indicators of OSA (AHI and ODI) were performed. As shown in **Figure** **1**A, higher ALT and AST levels were significantly associated with higher AHI and ODI. This finding suggests that poor liver function is associated with more severe OSA. Different death mechanisms may participate in this pathological process. Therefore, we tested the key proteins that represent the levels of different classical programmed deaths. IHC results indicated that only the level of GPX4, a biomarker of ferroptosis, was significantly lower in the liver tissues of patients with OSA (Figure 1B). The level of iron was higher in the liver tissues of patients with OSA, as shown by Prussian blue staining (Figure 1C). The level of MDA, which represents lipid peroxidation, was also higher in the OSA group (Figure 1D), whereas the level of the anti‐ferroptosis protein GSH was significantly lower in the OSA group (Figure 1E).

### 2.2 Chronic Intermittent Hypoxia Promoted NAFLD Progression in Mice

**Figure 2:**
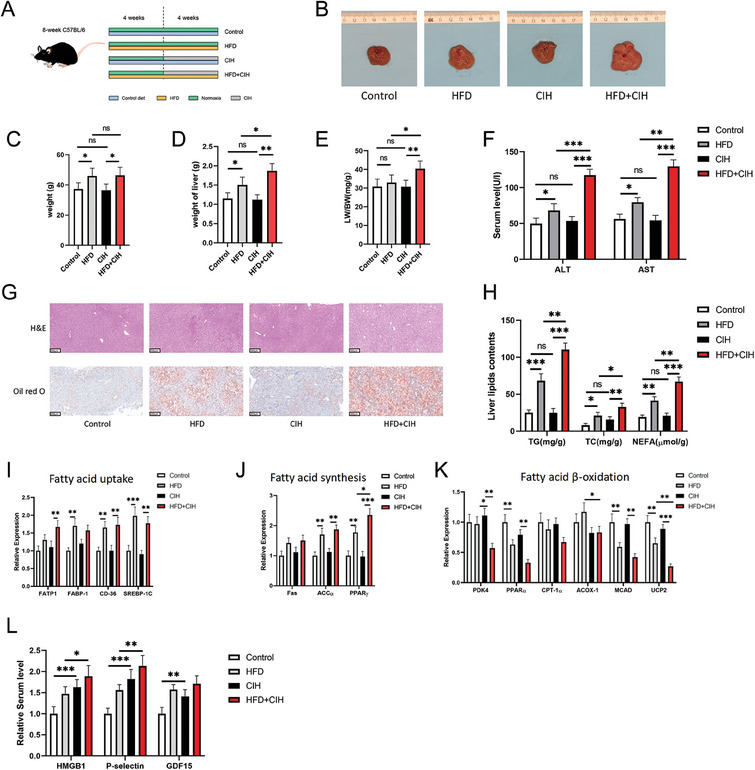
Chronic intermittent hypoxia promoted NAFLD progression in mice. A) Interventions for different groups of mice. B) Representative images of the livers from control, HFD, CIH, and HFD+CIH mice after eight weeks of intervention (*n* = 5 per group). C) Body weights of mice after modeling. D) Liver weight and E) LW/BW ratio of the four groups. F) Serum ALT and AST levels in the four groups. G) H&E staining and Oil red O staining of mouse liver tissues (scale bar, 100 µm). H) Mouse liver lipid levels: TG, TC, and NEFA levels. I) Transcription levels of fatty acid uptake, J) fatty acid synthesis, and K) fatty acid β‐oxidation‐related genes in mouse liver tissues. L) Serum HMGB1, P‐selectin, and GDF15 levels. All bar charts shown represent the mean ± SEM, ^*^ *p* < 0.05, ^**^ *p* < 0.01, ^***^ *p* < 0.001, ns, not significant, by ANOVA.

To clarify the effect of ferroptosis on OSA‐related NAFLD, we established OSA and NAFLD models in C57BL/6 mice and performed experiments in vivo (**Figure** **2**A). A direct‐view image demonstrated that the liver volume in the HFD group was significantly greater than that in the control group, and CIH significantly aggravated this HFD‐induced increase in liver volume, whereas CIH alone did not cause a significant change in liver volume (Figure 2B). The HFD significantly increased the body weight of mice after eight weeks of intervention; however, CIH did not significantly affect the body weight (Figure 2C). Figure 2D–F shows that CIH exacerbated the increase in liver weight, liver weight‐to‐body weight (LW/BW) ratio, and liver function indices (ALT and AST) caused by HFD. H&E staining showed that the liver tissue of the HFD group demonstrated increased steatosis and vacuoles; moreover, the integrity of the tissue structure was lost compared with that of the control group. HFD+CIH treatment aggravated these changes (Figure 2G). To determine the changes in lipid metabolism in the liver, we performed Oil red O staining and measured the liver lipid content. Oil red O staining showed that HFD led to browning of the liver tissue, and the staining in the HFD+CIH group was even more intense than that in the control group; however, CIH alone did not affect the staining (Figure 2G). The same trend was observed for the TG, TC, and NEFA levels (Figure 2H). HFD+CIH treatment had an evident effect on lipid metabolism in the liver; therefore, we assessed the lipid metabolism‐related genes. The results suggested that HFD‐induced lipid metabolism involved changes in fatty acid uptake, synthesis, and β‐oxidation gene transcription levels; promoted fatty acid uptake and synthesis; and inhibited β‐oxidation of lipids. CIH mainly affected the transcription levels of the PPARγ, ACOX1, and UCP2 genes and further aggravated the HFD‐induced lipid metabolism disorders (Figure 2I–K). As shown in Figure 2L, CIH also increased serum proinflammatory cytokine levels.

### 2.3 Inhibition of Ferroptosis Alleviated CIH‐Induced Liver Function Injury in HFD‐Fed Mice

**Figure 3:**
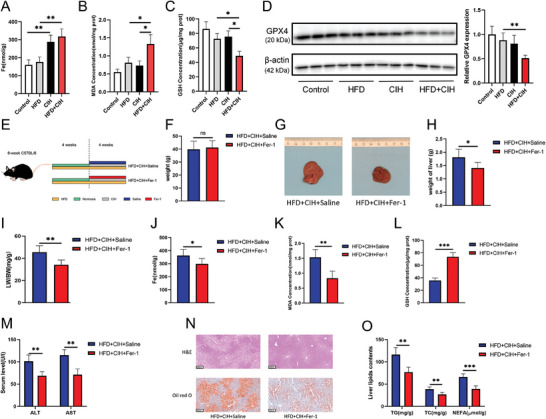
Inhibition of ferroptosis alleviated CIH‐induced liver function injury in HFD‐fed mice. A) Iron levels (liver iron content was normalized to liver mass), B) MDA, and C) GSH in mouse liver tissues from the control, HFD, CIH, and HFD+CIH groups (*n* = 5 per group). D) Western blotting was used to detect GPX4 levels in mouse liver tissues. E) Interventions for the different groups of mice. F) Weight, G) representative images, H) liver weight, and I) LW/BW ratio of the mice in HFD+CIH+saline and HFD+CIH+Fer‐1 groups. Ferroptosis indices showed the concentrations of J) iron, K) MDA, and L) GS in mouse liver tissues from the two groups. M) Mouse serum levels of ALT and AST in the two groups. N) H&E and Oil red O staining of mouse liver tissues (scale bar, 100 µm). O) Mouse serum TG, TC, and NEFA levels. All bar charts shown represent the mean ± SEM, ^*^ *p* < 0.05, ^**^ *p* < 0.01, ^***^ *p* < 0.001, A‐D by ANOVA, F‐O by *t*‐test.

Next, we examined the level of ferroptosis in the liver tissues of mice. As shown in **Figure** **3**A, CIH induced iron overload, and the iron content in the liver tissue of the CIH and HFD+CIH groups was significantly higher than that in the control and HFD groups. Moreover, the MDA level in the HFD+CIH group was significantly higher than that in the HFD group (Figure 3B). However, the concentration of GSH was significantly lower in the HFD+CIH group (Figure 3C). We then tested the level of GPX4 in the liver tissue, and observed that HFD+CIH treatment significantly reduced GPX4 protein levels (Figure 3D). These findings suggest that decreased GPX4 protein levels may play an important role in decreasing liver cell resistance to ferroptosis, thereby inducing hepatocyte ferroptosis and promoting NAFLD progression.

We investigated the role of ferroptosis in the progression of CIH‐induced NAFLD. The ferroptosis inhibitor, Fer‐1, was intraperitoneally injected into HFD‐fed mice subjected to CIH (Figure 3E). Four weeks later, no significant difference in body weight was observed between the two groups (Figure 3F). However, the volume and weight of the liver in the Fer‐1 group were significantly greater than those in the control group (Figure 3G,H). LW/BW was lower in the Fer‐1 group (Figure 3I). Determination of iron, MDA, and GSH levels in liver tissue verified that Fer‐1 successfully alleviated the increase in ferroptosis in liver tissue caused by HFD and CIH (Figure 3J–L). These findings were further confirmed by the lower ALT and AST levels in the livers of the Fer‐1 group compared with those of the control group (Figure 3M). H&E and Oil red O staining analyses showed that Fer‐1 alleviated the increase in hepatic lipid accumulation (Figure 3N). Consistently, the TG, TC, and NEFA levels decreased with Fer‐1 intervention (Figure 3O). These results suggest that the inhibition of ferroptosis by Fer‐1 alleviates liver function injury in HFD+CIH mice, suggesting an important role of ferroptosis in OSA‐related NAFLD.

### 2.4 Supernatants Derived from Macrophages under Intermittent Hypoxia Promoted Ferroptosis in Hepatocytes

**Figure 4:**
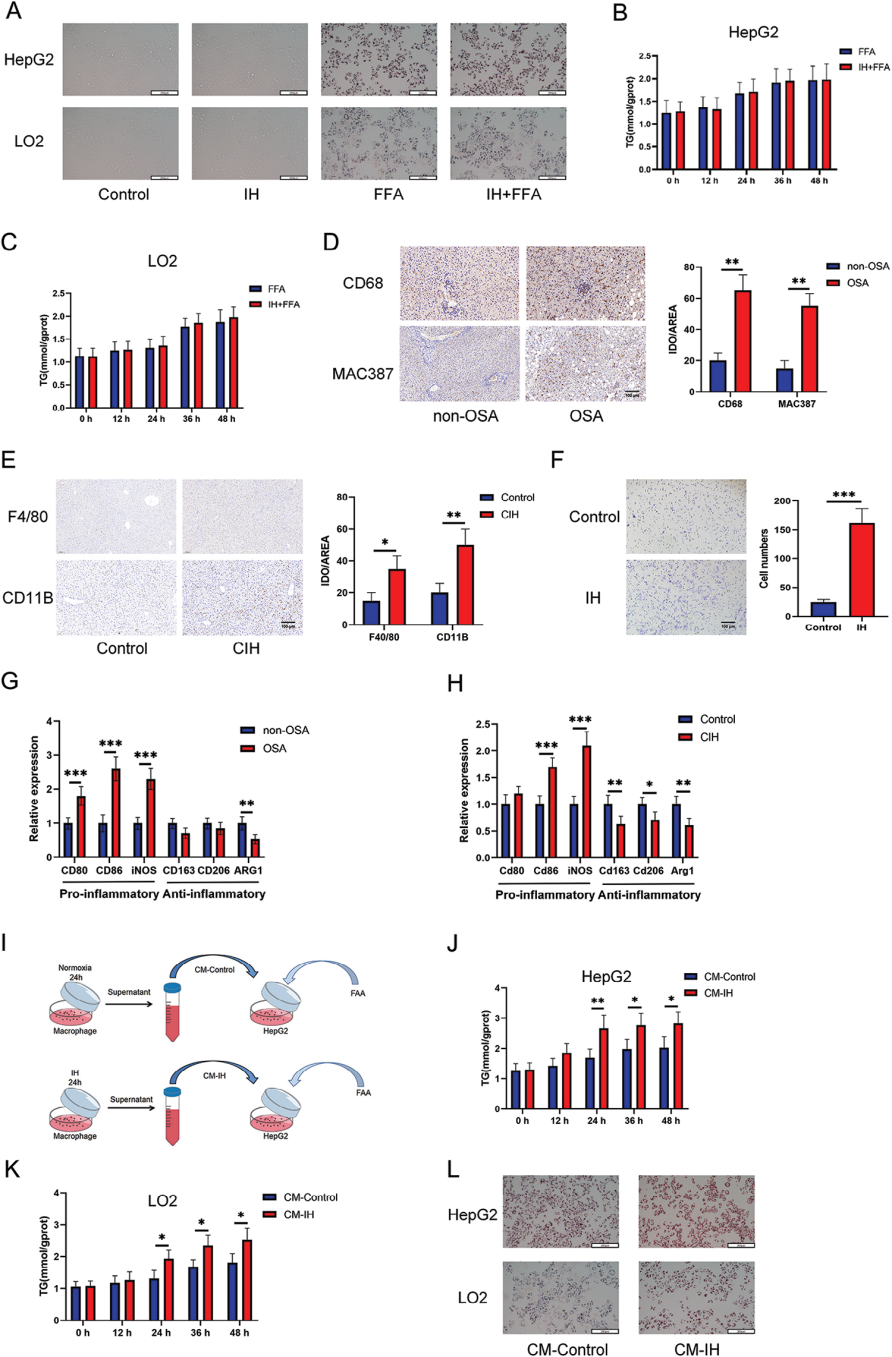
Supernatants derived from macrophages under IH promoted lipid accumulation in hepatocytes. A) Oil Red O staining of HepG2 and LO2 cells from the control, IH, FFA (1 × 10^−3^ m, 24 h), and IH+FFA groups (scale bar, 100 µm). B,C) TG levels in FFA‐ and IH+FFA‐treated hepatocytes over time. D) IHC was used to evaluate the levels of macrophage markers CD68 and MAC387 in liver tissues of patients with or without OSA (scale bar, 100 µm; non‐OSA = 10, OSA = 10). E) IHC was used to evaluate the levels of macrophage markers F4/80 and CD11B in the liver tissues of control and CIH groups (scale bar, 100 µm). F) Transwell assay of macrophages. G) Transcription levels of active macrophages markers in liver tissues of patients with or without OSA. H) Transcription levels of active macrophages markers in the liver tissues of control and CIH groups. I) Schematic diagram of the in vitro construction of cell model. J,K) TG levels in HepG2 and LO2 cells treated with CM‐Control or CM‐IH over time. L) Oil Red O staining of HepG2 and LO2 cells from the CM‐Control and CM‐IH groups (scale bar, 100 µm). All bar charts shown represent the mean ± SEM, ^*^ *p* < 0.05, ^**^ *p* < 0.01, ^***^ *p* < 0.001, by *t*‐test.

To explore the mechanism by which OSA promotes NAFLD progression, we exposed the cells to IH for 24 h to construct an OSA model in vitro and applied 1 × 10^−3^ m FFAs to the cells for 24 h to construct an NAFLD model in vitro. Surprisingly, IH did not increase the FFA‐induced lipid accumulation (**Figure** **4**A) or TG content in hepatocytes (Figure 4B,C). These findings suggested that IH does not directly promote NAFLD progression.

Studies have revealed that macrophage infiltration plays an important role in tissue damage induced by hypoxia–reperfusion, whose pathological phenomena are similar to those of IH in OSA.^[^ ^20^, ^21^ ^]^ Therefore, we examined macrophage infiltration into the liver tissues. Figure 4D shows increased macrophage infiltration in the liver tissue of patients with OSA. Increased macrophage infiltration was also observed in the liver tissues of the CIH‐treated mice (Figure 4E). Consistent with this, the conditioned medium of HepG2 cells under IH conditions significantly promoted macrophage migration (Figure 4F). Collectively, these findings suggest that IH, a characteristic of OSA, may play an important role in macrophage infiltration into the liver tissue. The qPCR results also showed that macrophages in the liver were activated into a proinflammatory phenotype in patients with OSA and CIH mice (Figure 4G,H). Hence, we examined its function. CM‐IH (Figure 4I) significantly increased TG content (Figure 4J,K) and Oil red O staining (Figure 4L).

**Figure 5:**
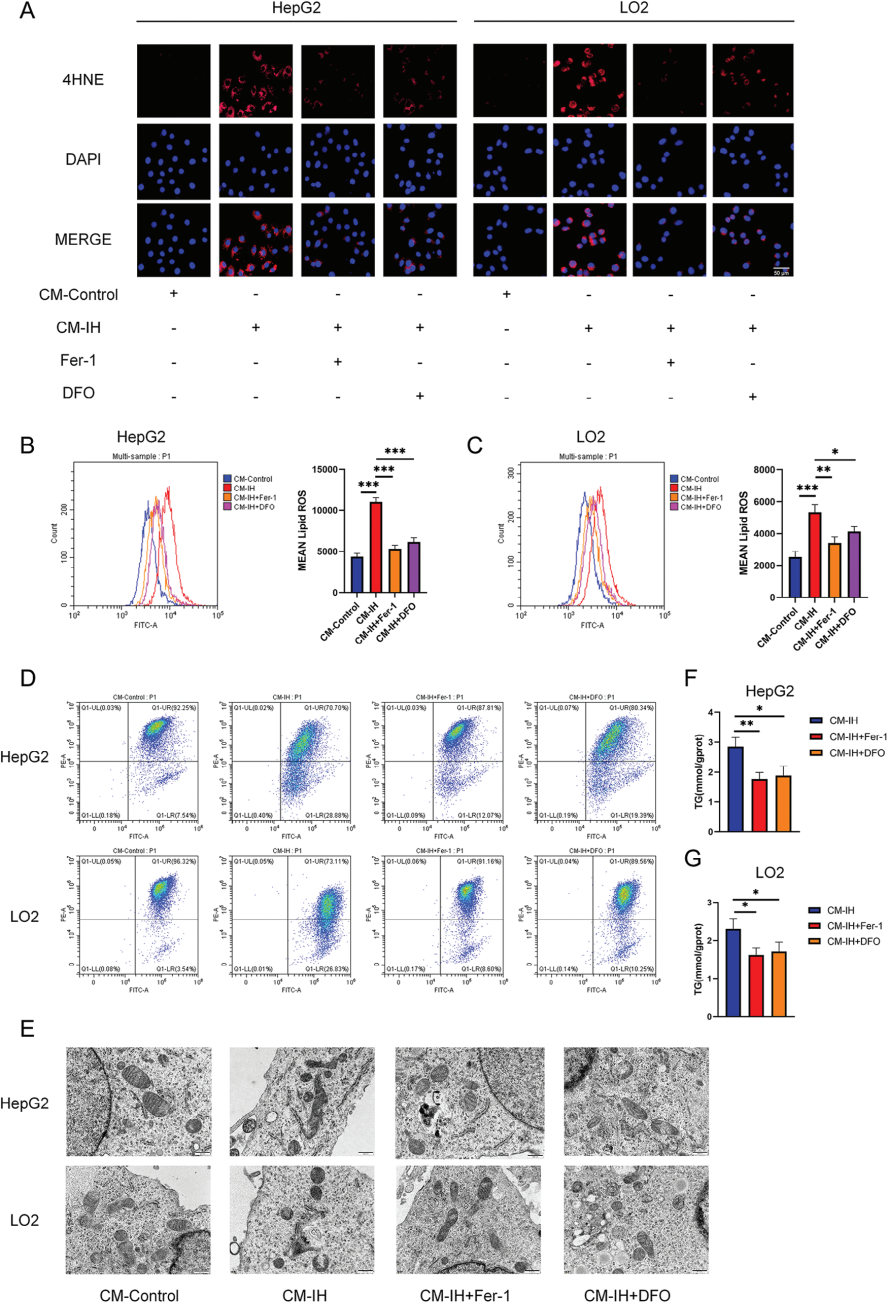
Supernatants derived from macrophages under IH promoted lipid accumulation in hepatocytes by inducing ferroptosis. A) Level of 4‐HNE in HepG2 (F) and LO2 (G) cells treated with Fer‐1 (10 × 10^−6^ m) or DFO (100 × 10^−6^ m) 24 h was determined using IF (scale bar, 100 µm). B,C) Level of lipid ROS determined using flow cytometry. D) Mitochondrial membrane potential was assessed using JC‐1 staining, which revealed mitochondria with high (increased PE) or low (increased FITC) membrane potential. E) Mitochondrial structure in HepG2 and LO2 cells under an electron microscope (scale bar, 500 nm). TG levels in F) HepG2 and G) LO2 cells treated with Fer‐1 or DFO. All bar charts shown represent the mean ± SEM, ^*^ *p* < 0.05, ^**^ *p* < 0.01, ^***^ *p* < 0.001, by ANOVA.

The effects of CM‐IH on hepatocyte ferroptosis were also evaluated. The levels of 4‐HNE (**Figure** **5**A) and lipid ROS (Figure 5B,C) were significantly increased in the CM‐IH group. The ferroptosis inhibitors Fer‐1 and DFO attenuated the increase in 4‐HNE and lipid ROS, and a decrease in the mitochondrial membrane potential was observed in the CM‐IH group (Figure 5D). CM‐IH also induced a mitochondrial ferroptosis phenotype characterized by membrane thickening, disappearance of cristae, and rupture of mitochondria (Figure 5E). Furthermore, inhibitors of ferroptosis could alleviate mitochondrial damage. Fer‐1 and DFO also alleviated the CM‐IH‐led increase in TG content (Figure 5F,G). Taken together, these data demonstrate that macrophages play a role in OSA‐related NAFLD via ferroptosis.

### 2.5 IL‐6 Derived from Macrophages under IH Induced Lipid Accumulation and Ferroptosis in Hepatocytes

**Figure 6:**
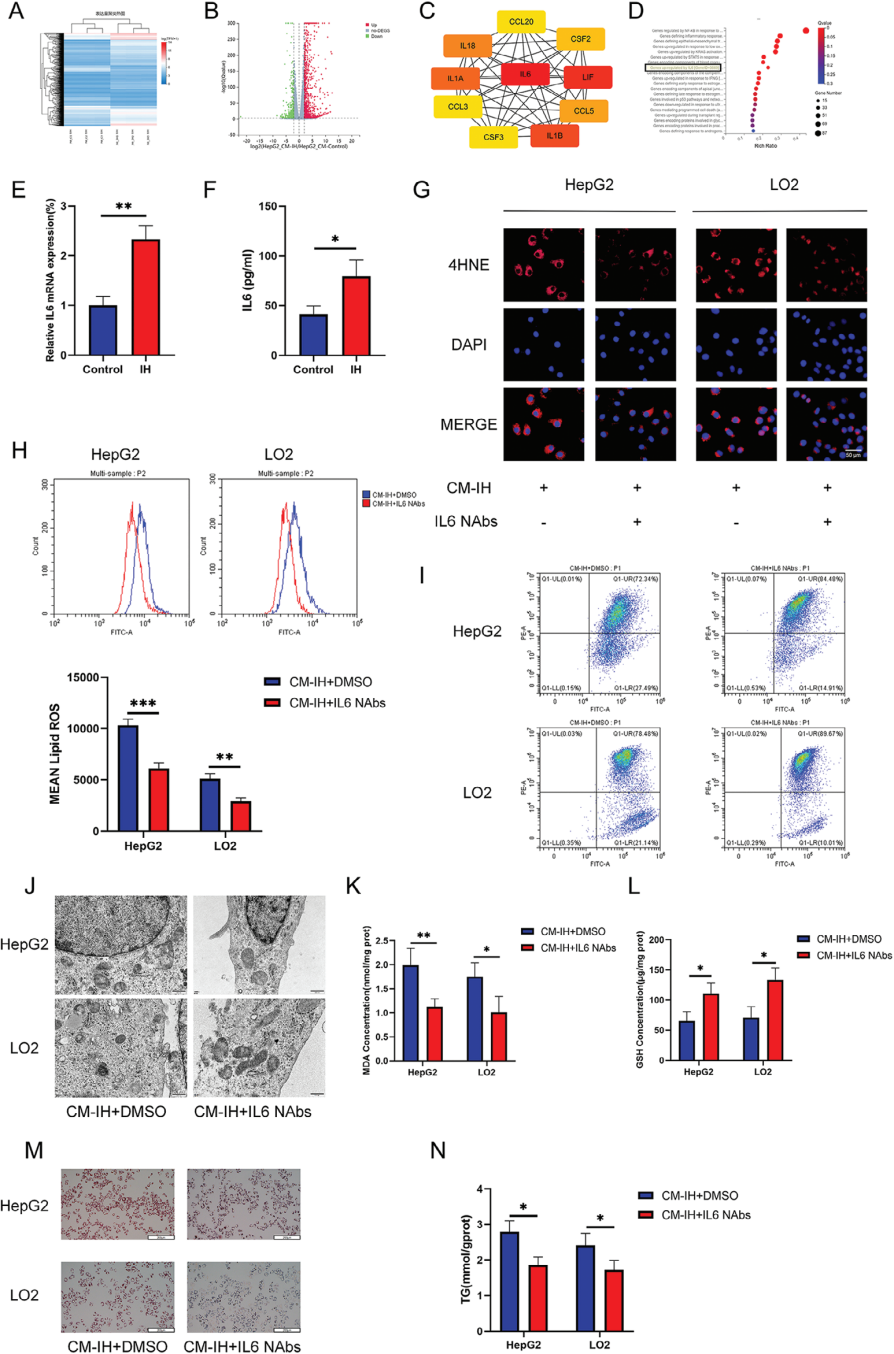
IL‐6 derived from macrophages under IH induced ferroptosis and lipid accumulation in hepatocytes. A–D) RNA‐seq data of HepG2 cells treated with CM‐control or CM‐IH (24 h). A) Heatmap, B) volcano map, C) PPI analysis, and D) enrichment analysis of DEGs. E) IL6 mRNA level in macrophages evaluated by q‐PCR. F) IL6 concentration in the macrophage supernatant determined by ELISA. G) Level of 4‐HNE determined using IF (scale bar, 100 µm). H) Level of lipid ROS determined using flow cytometry. I) Mitochondrial membrane potential was assessed using JC‐1 staining, which revealed mitochondria with high (increased PE) or low (increased FITC) membrane potential. J) Mitochondrial structure in HepG2 and LO2 cells under an electron microscope (scale bar, 500 nm). Levels of K) MDA and L) GSH in hepatocytes. M) Oil Red O staining of HepG2 and LO2 cells from the DMSO and IL6 NAbs groups (200 ng mL^−1^, 24 h). N) TG level in HepG2 or LO2 cells treated with DMSO or IL6 NAbs. All bar charts shown represent the mean ± SEM, ^*^ *p* < 0.05, ^**^ *p* < 0.01, by *t*‐test.

To evaluate the underlying mechanisms, we performed transcriptome sequencing of HepG2 cells exposed to CM‐control or CM‐IH. **Figure** **6**A shows a heatmap of gene expression and Figure 6B shows the DEGs between the two groups. IL6 was identified as the core protein through protein–protein interaction (PPI) analysis (Figure 6C). Consistently, DEG enrichment analysis indicated an important role for IL6 (Figure 6D). Therefore, we focused on IL6 in the follow‐up study.

Both IL6 mRNA level in macrophages and the level of IL6 in the supernatant increased after IH intervention (Figure 6E,F). IL6 NAbs intervention significantly reduced the levels of 4‐HNE (Figure 6G) and lipid ROS (Figure 6H). Both mitochondrial membrane potential (Figure 6I) and transmission electron microscopy (Figure 6J) results indicated that IL6 NAbs intervention alleviated the ferroptotic phenotype of the mitochondria. A decrease in MDA levels was observed in the CM‐IH+IL6 NAbs group (Figure 6K), whereas the level of GSH increased in the CM‐IH+IL6 NAbs group (Figure 6L). As shown in Figure 6M,N, the addition of IL6 NAbs reduced lipid accumulation induced by CM‐IH.

### 2.6 IL6‐induced Degradation of GPX4 Protein in Hepatocytes was Dependent on MARCH3

**Figure 7:**
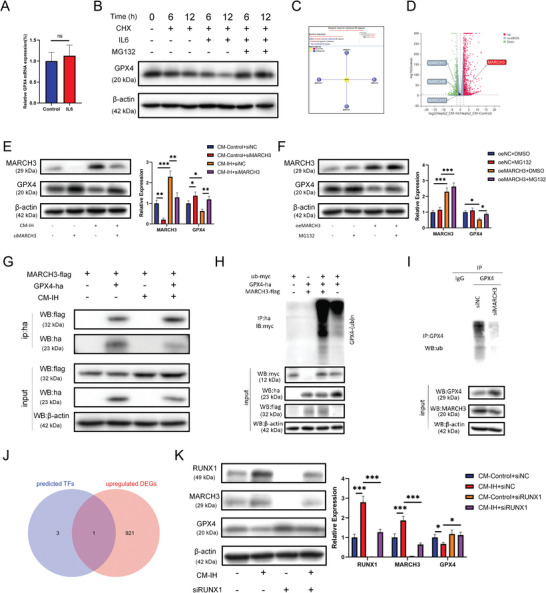
IL6‐induced degradation of GPX4 protein in hepatocytes was dependent on MARCH3. A) GPX4 mRNA levels in HepG2 cells evaluated by q‐PCR. B) GPX4 protein levels in HepG2 cells determined by western blot analysis (20 × 10^−6^ m MG132, 100 µg mL^−1^ CHX). C) Potential E3 ligases of GPX4 predicted by UbiBrowser. D) Transcription levels of MARCH1, MARCH3, MARCH8, and MARCH11 determined via RNA‐seq in HepG2 cells. E,F) Levels of GPX4 and MARCH3 in HepG2 cells treated with different interventions were evaluated by western blot analysis. G) Interaction between MARCH3 and GPX4 was confirmed by co‐IP. GPX4 ubiquitination level in cells with H) exogenous overexpression or I) endogenous knockdown of MARCH3. J) Levels of nGPX4(nucleus GPX4), cGPX4(cytoplasm GPX4) and mGPX4(mitochondria GPX4) in HepG2 cells treated with different interventions were evaluated by western blot analysis. K) Intersection of DEGs and predicted transcription factors targeting MARCH3 genes was shown using a Venn diagram. L) Levels of RUNX1, MARCH3, and GPX4 in HepG2 cells treated with CM‐IH and siRUNX1. All bar charts shown represent the mean ± SEM, ^*^ *p* < 0.05, ^**^ *p* < 0.01, ^***^ *p* < 0.001, ns, not significant, A by *t*‐test, B‐K by ANOVA.

We examined the effect of IL6 on GPX4 transcription in hepatocytes. The qPCR results indicated no significant difference in GPX4 transcript levels in response to IL6 intervention (**Figure** **7**A). However, IL6 significantly decreased the GPX4 protein levels in hepatocytes in vitro (Figure 7B). Therefore, we speculated that the decrease in IL6‐mediated GPX4 protein levels may be caused by increased protein degradation. Thus, a proteasome inhibitor, MG132, was added to the medium supplemented with IL6. As shown in Figure 7B, MG132 rescued the decrease in GPX4 protein levels induced by IL6. These data suggest that ubiquitination‐mediated degradation may be the potential mechanism by which IL6 decreased GPX4 protein levels. To further clarify the key proteins regulating the increase in GPX4 ubiquitination‐mediated degradation, we used UbiBrowser to predict the E3 ligases of GPX4. A total of four E3 ligases were screened: MARCH1, MARCH3, MARCH8, and MARCH11 (Figure 7C). After combining the DEGs identified by RNA‐Seq (Figure 7D), MARCH3 was identified as the most promising target. As shown in Figure 7E, CM‐IH increased MARCH3 levels, and siMARCH3 alleviated the CM‐IH‐induced downregulation of GPX4. Furthermore, the oeMARCH3‐induced decrease in GPX4 expression was suppressed by MG132 treatment (Figure 7F). These results suggest that increased MARCH3 expression promotes GPX4 ubiquitination and degradation. We then transfected MARCH3‐Flag and GPX4‐HA overexpression plasmids into 293T cells and confirmed the interaction between MARCH3 and GPX4 by co‐IP (Figure 7G). Next, we determined whether MARCH3 influences GPX4 ubiquitination under IH conditions. Exogenous MARCH3 overexpression strongly increased the ubiquitination level of GPX4 in HepG2 cells (Figure 7H). siMARCH3 decreased the ubiquitination level of endogenous GPX4 protein in HepG2 cells (Figure 7I), suggesting that MARCH3 mediates the ubiquitination and degradation of GPX4 under IH. We further performed subcellular fractionation to separate the cellular components. Western blotting showed that the subcellular localization of GPX4 degradation were localized to cytoplasm and mitochondria (Figure 7J). We predicted the potential transcription factors for MARCH3 using a transcription factor prediction website and intersected these with the DEGs from transcriptome sequencing. RUNX1 was ultimately identified as the most likely transcription factor regulating MARCH3 (Figure 7K), and the regulation of MARCH3 by RUNX1 was confirmed by western blotting (Figure 7L).

### 2.7 Knockdown of MARCH3 Alleviated Ferroptosis and Lipid Accumulation Induced by CM‐IH

**Figure 8:**
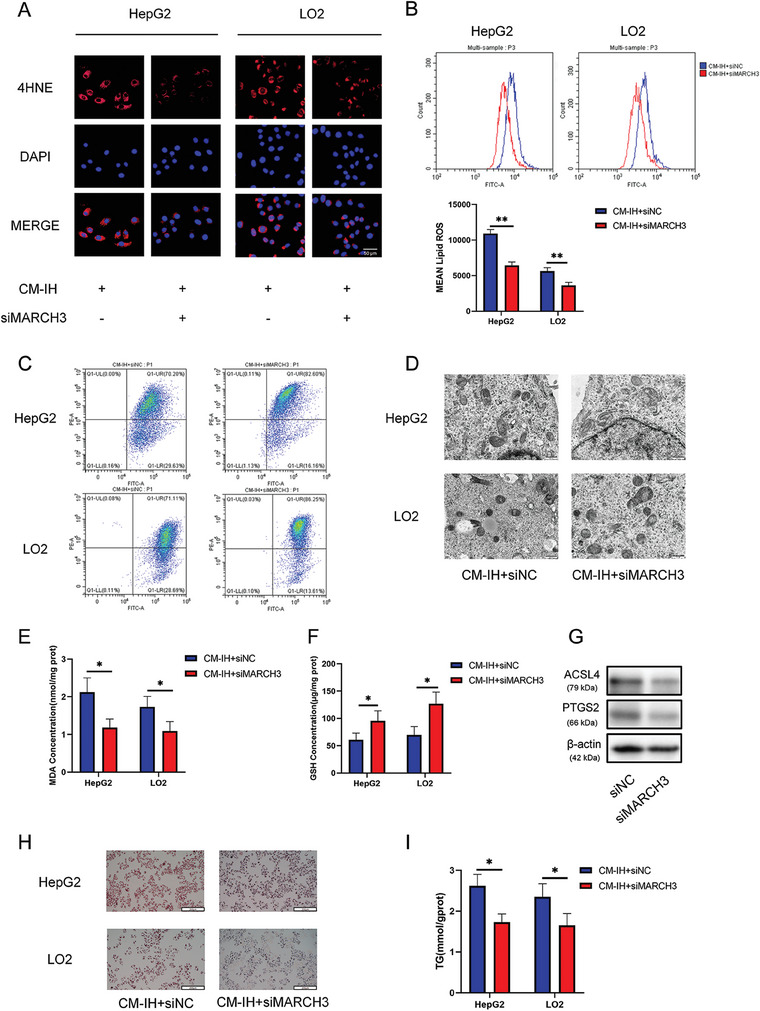
Knockdown of MARCH3 alleviated CM‐IH‐induced ferroptosis and lipid accumulation in hepatocytes. A) Level of 4‐HNE determined using IF (scale bar, 100 µm). B) Level of lipid ROS determined using flow cytometry. C) Mitochondrial membrane potential was assessed by JC‐1 staining, which revealed mitochondria with high (increased PE) or low (increased FITC) membrane potential. D) Mitochondrial structure in HepG2 and LO2 cells under an electron microscope (scale bar, 500 nm). Levels of E) MDA and F) GSH in hepatocytes. G) Expression of ACSL4 and PTGS2 were detected by western blot analysis. H) Oil Red O staining of HepG2 and LO2 cells from the siNC and siMARCH3 groups (scale bar, 100 µm). I) TG levels in HepG2 or LO2 cells treated with siNC or siMARCH3. All bar charts shown represent the mean ± SEM, ^*^ *p* < 0.05, ^**^ *p* < 0.01, by *t*‐test.

To further evaluate the effects of MARCH3, we examined the ferroptosis phenotype and lipid accumulation. MARCH3 knockdown alleviated the changes in 4‐HNE and lipid ROS levels (**Figure** **8**A,B) and rescued the changes in mitochondrial structure (Figure 8C,D). These results suggested that MARCH3 knockdown alleviated CM‐IH‐induced hepatocyte ferroptosis. This was further confirmed by the decrease in MDA levels and increase in GSH levels (Figure 8E,F). And Figure 8G showed that MARCH3 knockdown decreased ferroptosis markers levels. Moreover, MARCH3 knockdown reduced the CM‐IH‐induced lipid accumulation (Figure 8H,I).

### 2.8 Knocking Down MARCH3 Alleviated the Development of CIH‐Induced NAFLD In Vivo

**Figure 9:**
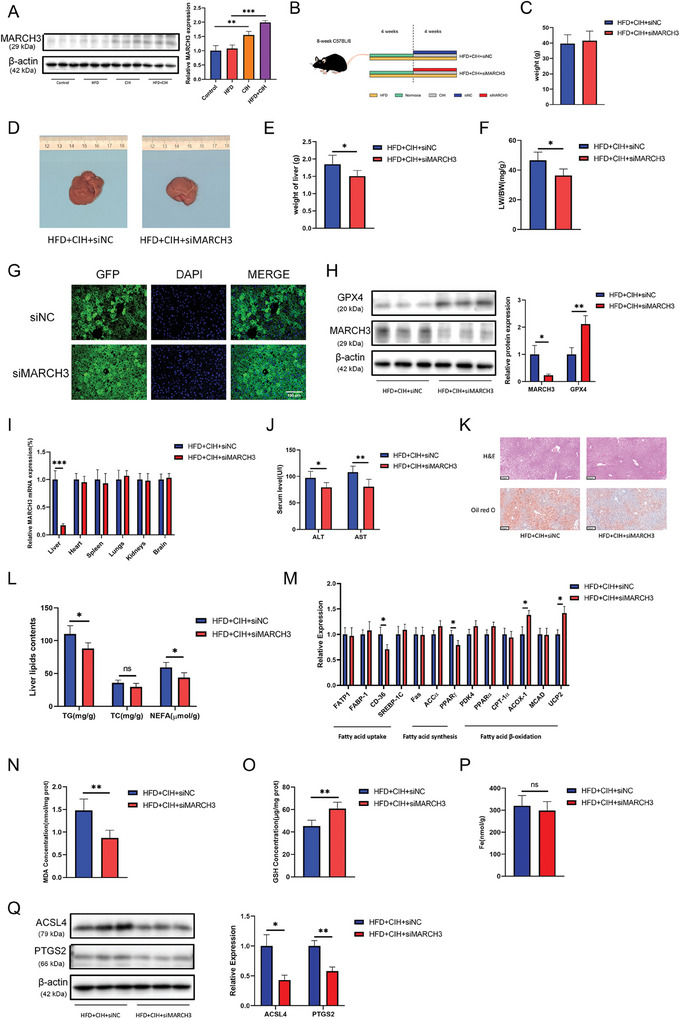
Knockdown of MARCH3 alleviated the development of CIH‐induced NAFLD in vivo. A) MARCH3 protein levels in mouse liver tissues were tested via western blot analysis (*n* = 5 per group). B) Schematic diagram of the interventions used in the different groups of mice. C) Weight of the mice after the intervention. D) Representative images, E) liver weight, and F) LW/BW ratios of the mouse livers. G) Expression of the viral vector fluorescent protein was detected by immunofluorescence. H) Levels of MARCH3 and GPX4 in mouse liver tissues. I) Transcription levels of MARCH3 in different mouse tissues. J) Serum ALT and AST levels. K) H&E staining and Oil red O staining of mouse liver tissues (scale bar, 100 µm). L) Mouse liver lipid contents: TG, TC, and NEFA levels. M) Transcription levels of fatty acid metabolism‐related genes. Levels of N) MDA, O) GSH, and P) iron in mouse livers. Q) Expression of ACSL4 and PTGS2 were detected by western blot analysis. All bar charts shown represent the mean ± SEM, ^*^ *p* < 0.05, ^**^ *p* < 0.01, ^***^ *p* < 0.001, ns, not significant, by *t*‐test.

First, we tested MARCH3 protein levels in the liver tissues of mice harvested as described in Result 2, and the results showed that MARCH3 levels were upregulated in the CIH and HFD+CIH groups (**Figure** **9**A). To evaluate the function of the construct in vivo, siMARCH3 or siNC liver‐specific adeno‐associated viruses were injected into mice through the caudal vein (Figure 9B). After modeling, no significant changes in weight were observed between the two groups (Figure 9C). However, the liver size was smaller, as visible by the naked eye, in the HFD+CIH+siMARCH3 group (Figure 9D), and siMARCH3 decreased the liver weight (Figure 9E) and LW/BW ratio (Figure 9F). Figure 9G shows the adeno‐associated virus‐infected mouse livers from different groups, and Figure 9I shows March3 is specifically targeted in the liver tissue. The effect of the adeno‐associated virus on MARCH3 knockdown in the liver tissues of mice was determined by western blotting (Figure 9H). Additionally, GPX4 was upregulated in the HFD+CIH+siMARCH3 group. MARCH3 knockdown decreased ALT and AST levels in HFD+CIH mice (Figure 9J). As expected, MARCH3 knockdown improved the structure of liver tissue and decreased the amount of Oil red O staining (Figure 9K). The TG and NEFA levels in the liver were also decreased in the siMARCH3 group (Figure 9L). Collectively, these data indicate that downregulation of MARCH3 improves liver function and lipid metabolism. Transcript levels of lipid metabolism genes confirmed the improvement in lipid metabolism induced by siMARCH3 intervention (Figure 9M). Consistent with these findings, siMARCH3 decreased ferroptosis in the liver tissues of HFD+CIH mice, decreased MDA concentrations (Figure 9N), and rescued GSH levels (Figure 9O). Moreover, ferroptosis markers decreased significantly in siMARCH3 mice(Figure 9Q). However, no significant difference in iron concentration was detected between the two groups (Figure 9P).

## 3 Discussion

Obstructive sleep apnea (OSA) is the most common sleep‐disordered breathing, affecting approximately one in seven adults worldwide. OSA is characterized by repeated hypoxia/reoxygenation during sleep. Thus, chronic intermittent hypoxia (CIH) is one of the most common pathological features of OSA, which leads to multiple organ and systemic complications due to oxidative stress and systemic inflammation.^[^ ^1^ ^]^ The liver, an important metabolic organ, is sensitive to hypoxic injury, which may be associated with chronic liver metabolic abnormalities and NAFLD caused by OSA.^[^ ^5^ ^]^ In this study, we found that the liver function indices, ALT and AST, were positively correlated with OSA severity in patients with NAFLD. In vitro and in vivo experiments have suggested that the induction of lipid peroxidation and ferroptosis in hepatocytes may be the mechanism underlying the exacerbation of NAFLD induced by IH.

Macrophage infiltration plays an important role in various types of OSA‐related organ injuries. Studies have reported that the risk of cardiovascular diseases is significantly increased in patients with OSA because macrophage infiltration causes vascular endothelial damage and dysfunction.^[^ ^22^ ^]^ Increased macrophage infiltration induced by IH promotes the occurrence and progression of cardiovascular diseases via a proinflammatory cascade reaction.^[^ ^23^ ^]^ Significantly increased infiltration of macrophages was also observed in the adipose tissue of patients with OSA, accompanied by changes in the polarization phenotype of macrophages, mostly from M2 to M1. Infiltrating macrophages secrete numerous inflammatory factors, resulting in adipose tissue inflammation and metabolic disorders. These effects can lead to insulin resistance and diabetes.^[^ ^20^ ^]^ Macrophage infiltration is also associated with OSA‐related kidney injury.^[^ ^21^ ^]^ Macrophages play an important role in the pathogenesis of OSA‐related diseases. In this study, we found that macrophage infiltration significantly increased in the liver tissues of patients with OSA and mice exposed to IH, which was confirmed by the increased migration ability of macrophages induced by the hepatocyte supernatant under IH conditions. Increased macrophage infiltration promotes NAFLD progression. Furthermore, through RNA‐seq of hepatocytes and ELISA of the macrophage supernatant, we found that increased IL6 in the supernatant may be the key factor leading to lipid peroxidation and abnormal lipid accumulation in hepatocytes.

IL6 is produced and secreted by various cells including macrophages. Its role in the regulation of inflammation has been widely studied. IL6 has dual effects on inflammation; it plays an anti‐inflammatory role at a low dose during the initial stage of injury, whereas it acts as a proinflammatory cytokine at a high dose.^[^ ^24^ ^]^ The level of IL6 increases with the progression of NAFLD, and IL6 content has been shown to be associated with the severity of NAFLD.^[^ ^25^, ^26^ ^]^ IL6 inhibition alleviates NAFLD progression.^[^ ^27^ ^]^ In our study, the macrophages secreted increased level of IL6. RNA‐seq of HepG2 cells confirmed the important role of IL6, which was further confirmed by the finding that IL6 NAbs attenuate OSA‐induced NAFLD progression. We also found that IL6 induced lipid peroxidation and ferroptosis in hepatocytes, which may be important targets for IH to promote the development of NAFLD. In previous studies, IL6 has been shown to exert its effects mainly via the JAK2/STST3, MAPK, and PI3K/AKT pathways.^[^ ^28^ ^]^ However, how IL6 regulates ferroptosis in hepatocytes under IH conditions has not yet been determined. Using an in vitro cell model, we found that increased IL6 derived from CM‐IH induced a significant decrease in GPX4 protein levels in hepatocytes; however, there was no significant difference at the transcriptional level. The proteasome inhibitor MG132 rescued the decrease in GPX4 protein levels induced by IL6, which suggested that ubiquitination and degradation play important roles in the IL6‐induced decrease in the levels of GPX4 protein. Furthermore, MARCH3 was selected as the most potent E3 ligase for GPX4.

The membrane‐associated RING‐CH (MARCH) family of E3 ubiquitin ligases has attracted widespread attention in recent years. Currently, 11 proteins have been identified (MARCH1‐11). These proteins share an N‐terminal RING finger domain, also known as the ring domain. This structure is important for these proteins because it functions as an E3 ligase and can bind to the E2 ubiquitin‐binding enzyme to mediate the ubiquitination‐mediated degradation of proteins.^[^ ^29^ ^]^ Recently, the role of the MARCH family members in regulating immunity and inflammation has attracted widespread attention. MARCH3 is a transmembrane protein of the MARCH family, which is located mainly in the lysosomal and endosomal membranes, but is also distributed in the cytoplasmic membrane. Previous studies have shown that MARCH3 is involved mainly in protein ubiquitination, vesicle transport, and immune system regulation.^[^ ^30^ ^]^ Studies have reported regulatory effects of MARCH3 on inflammatory responses. MARCH3 alleviated damage to the inflammatory response via polymerized ubiquitination modified and inhibited the IL‐1β‐induced inflammatory response through lysosomal degradation.^[^ ^31^ ^]^ A previous study also reported that MARCH3 induces the ubiquitination‐mediated degradation of IL‐3R, and the loss of MARCH3 enhances the expression of downstream inflammatory genes induced by IL3, thus promoting the inflammatory response.^[^ ^32^ ^]^ In the present study, the upregulation of MARCH3 was induced by an increase in IL6 in the macrophage supernatant under IH, and the upregulation of MARCH3 in CIH mouse liver tissues was attenuated by IL6 NAbs. MARCH3 acts as an E3 ligase that regulates GPX4 ubiquitination and degradation, thereby promoting ferroptosis in hepatocytes. Although MARCH3 has been shown to inhibit inflammation, MARCH3 overexpression is an important regulatory factor that promotes the progression of OSA‐related NAFLD. In vivo, MARCH3 knockdown inhibited ferroptosis and liver injury in liver tissue in HFD+CIH mice. These results suggested that MARCH3 is an important target for the prevention and treatment of OSA‐related NAFLD.

## 4 Experimental Section

### Patients and Specimens

The participants in this study were patients with obesity scheduled to undergo metabolic surgery at the Weight Loss and Metabolic Disease Surgery Center of the Zhongnan Hospital of Wuhan University from June 2020 to June 2021. The study included only those patients who met the requirements for weight‐loss‐related surgery and signed an informed consent form. All participants underwent overnight polysomnography (PSG). The exclusion criteria for patients were as follows: ① had other sleep‐related diseases (central sleep apnea, neuromuscular diseases, etc.); ② had a history of cranial trauma or neurological or psychiatric diseases; ③ had previous OSA‐related treatment; ④ had a history of infectious diseases; ⑤ had malignant tumors or acute and chronic renal dysfunction; ⑥ had alcoholic liver disease; ⑦ had viral hepatitis; ⑧ had drug‐induced liver disease; ⑨ had other diseases that can cause fatty liver disease, such as autoimmune liver disease; and ⑩ had iron metabolism disorder‐related diseases (hereditary hemochromatosis) or consumed iron supplements. Ultimately, 270 patients containing 252 OSA patients were included in this study. The research protocol was approved by the Ethics Committee of Zhongnan Hospital of Wuhan University (Ethics number: 2019021), and all patients included in the study signed an informed consent form. PSG data were recorded using Embletta Gold software (Embla Systems, Inc., Broomfield, CO, USA). Two experienced PSG technicians independently analyzed all the data obtained during a sleep period according to the standards of the American Academy of Sleep Medicine. The presence and severity of OSA were assessed based on the number of apnea and hypopnea events per hour (AHI). An AHI of ≥ 5 was considered to indicate OSA. The oxygen desaturation index (ODI) was defined as the number of times per hour of sleep that the blood oxygen level decreased by ≥4% from the baseline. Liver tissues were obtained during weight‐loss surgery by the same surgeon, ensuring the same sampling location. After washing with saline, a portion of the tissue was immediately fixed with 4% paraformaldehyde for subsequent experiments.

### Cell Culture

The HepG2, LO_2_, and THP1 cell lines were purchased from the Medical Science Research Center, Zhongnan Hospital of Wuhan University, and maintained in high‐glucose DMEM (Gibco, USA) or RPMI 1640 (Gibco, USA) supplemented with 10% fetal bovine serum (FBS; Cellmax, China). All media were supplemented with 100 U L^−1^ penicillin and 100 mg L^−1^ streptomycin (Beyotime, China). The cells were cultured in a humidified incubator in a 5% CO_2_ atmosphere at 37 °C. The IH model was generated using a hypoxia chamber (5% O_2_ for 35 min, 21% O_2_ for 25 min; one cycle per hour). Cell steatosis was induced by the addition of 1 × 10^−3^ m free fatty acids (FFAs, OA: PA, 2:1, Sigma‐Aldrich, USA) for 24 h, and FFA‐free bovine serum albumin was added at an equal final concentration to the control cells. THP‐1 cells were treated with 100 ng mL^−1^ PMA (MCE, China) for 48 h to induce macrophage differentiation. After 24 h of IH or normoxia culture, the supernatant was collected and used as the conditioned medium (CM‐control or CM‐IH). IL6 neutralizing antibodies (IL6 NAbs, 200 ng mL^−1^) (SinoBiological, China) were added to CM‐IH to inhibit IL6 in the CM‐IH+IL6 NAbs group.

### Transwell Migration Assay

Macrophagocytes were seeded in the upper transwell chambers and cultured in 100 µL RPMI 1640 without FBS. The chambers were subsequently placed in 24‐well plates supplemented with 700 mL conditioned medium. After 48 h, the membranes of the chambers were swabbed clean, and the migrated cells were stained with 0.3% crystal violet in ethanol. Three random fields in the chambers were examined by microscopy.

### Construction of MARCH3 Knockdown or GPX4 Overexpressed HepG2 Cell Lines

A small interfering ribonucleic acid (siRNA) targeting the mRNA sequence of MARCH3 (siMARCH3) was transfected into HepG2 cells using Lipofectamine 3000 (Thermo Fisher Scientific, USA). The GPX4 overexpression and negative control (NC) vectors synthesized by GeneCopoeia (Guangzhou, China) were transfected into HepG2 cells using Lipofectamine 3000 (Thermo, USA) according to the manufacturer's instructions.

### Animal Model

The high‐fat diet (HFD) mouse is a common animal model of NAFLD. In this study, C57BL/6 mice were fed D12492 HFD (Xietong, China) to establish an animal model of NAFLD. Chronic intermittent hypoxia (CIH) was induced after the mice were fed a high‐fat or conventional diet for four weeks. During the modeling period (12 h from 8 A.M. to 8 P.M. daily), the mice were placed in an animal incubator (Oxycyler Model A48, USA) with IH. The IH period included injection of nitrogen into the incubator for 1 min to reduce the oxygen concentration to 10%, which was maintained for 1 min. Oxygen was injected into the incubator for 1 min to increase the oxygen concentration to 20.9%, which was maintained for 1 min before the next intermittent anoxic cycle. In the control group, only air was injected into the incubator. The total CIH model duration was four weeks. Mice in the Fer‐1 intervention group were intraperitoneally injected with 1 mg kg^−1^ Fer‐1, and mice in the control group were intraperitoneally injected with the same volume of normal saline before daily IH modeling. For AAV9 transduction, AAV9‐MARCH3‐Control and AAV9‐MARCH3‐RNAi were purchased from the Genechem company (Shanghai, China) and injected via the tail vein (200 µL per mouse, virus titer: 4.61E+12 v.g. mL^−1^) before IH induction. Animal studies were approved by IACUC of Wuhan University Center for Animal Experiment (NO.WP20220036).

### RNA Extraction and Quantitative RT‐PCR

Total RNA was isolated using TRIzol reagent (Thermo Fisher Scientific, USA). The RNA quality and quantity were evaluated using a NanoDrop 3300 spectrophotometer (Thermo Fisher Scientific, USA). Reverse transcriptase (Vazyme, China) was used for reverse transcription. SYBR Green qPCR SuperMix‐UDG (Thermo Fisher Scientific, USA) was utilized to conduct quantitative real‐time PCR. GAPDH was used as the normalization control. At least three replicates were analyzed for each sample. The primers used are listed in Table S1, Supporting Information‐Primers.

### Western Blotting

Nuclear, cytoplasmic and mitochondria proteins were separated by Nuclear and Cytoplasmic Protein Extraction Kit (Beyotime, China) and Cell Mitochondria Isolation Kit (Beyotime, China). Then protein was extracted using RIPA lysis buffer (Beyotime, China) containing phosphatase and protease inhibitors. A BCA Protein Assay Kit (Thermo Fisher Scientific, USA) was used to quantify protein concentration. Equal amounts of protein (20 µg) were loaded and separated using 10% SDS‒PAGE and subsequently transferred onto PVDF membranes (Sigma‐Aldrich, USA). All the membranes were blocked with 5% skim milk for 1 h. The membranes were incubated overnight with primary antibodies at 4 °C. The next day, the membranes were incubated at room temperature for 2 h with horseradish peroxidase‐conjugated secondary antibodies (CST, USA). The protein bands were visualized using an enhanced chemiluminescence (ECL) detection kit (Beyotime, China). Relative protein levels were analyzed using the ImageJ software. β‐actin (Proteintech, China) was used as the internal control. The primary antibodies used were GPX4 (Proteintech, China), MARCH3 (Abmart, China), FLAG (ABclonal, China), HA (ABclonal, China), Myc (ABclonal, China), Ub (ABclonal, China), RUNX1 (ABclonal, China), ACSL4 (Proteintech, China) and PTGS2 (Proteintech, China).

### Assays for MDA, GSH, Fe, TG, NEFA, TC, ALT, AST, IL6, HMGB1, P‐Selectin, and GDF15 Level

The MDA, GSH, Fe, TG, NEFA, TC, ALT, AST, and IL6 levels were tested by using an MDA assay kit (Solarbio, China), a GSH assay kit (Solarbio, China), an iron colorimetric assay kit (Applygen, China), a TG assay kit (Njjcbio, China), an NEFA assay kit (Njjcbio, China), a TC assay kit (Njjcbio, China), an ALT assay kit (Njjcbio, China), an AST assay kit (Njjcbio, China), an IL6 assay kit (Neobioscience, China), an HMGB1 assay kit (Neobioscience, China), an P‐selectin assay kit (Neobioscience, China) and an GDF15 assay kit (Neobioscience, China), respectively, according to the manufacturer's instructions.

### Lipid ROS Assay

The molecular probe BODIPY 581/591 C11 (Thermo Fisher Scientific, USA) was used to measure the lipid ROS. The hepatocytes were incubated with the kit reagent at a working concentration of 5 × 10^−6^ m for 30 min in the dark. Lipid ROS levels were subsequently determined using flow cytometry (Beckman, USA).

### Mitochondrial Membrane Potential Assay

Mitochondrial membrane potential was assayed using the fluorescent probe JC1 (MCE, China). Add 10 µL JC‐1 (200 × 10^−6^ m) per well to make the final concentration at 2 × 10^−6^ m. Incubate cells at 37 °C, 5% CO2, for 20 min. Then the sample was analyzed by flow cytometry (Beckman, USA).

### RNA Sequencing

The sequencing samples used were CM‐IH‐ and CM‐control‐treated hepatocytes. After obtaining lysate samples from three independent experiments, the samples were subjected to BGI (Shenzhen, China) for transcriptome sequencing. The genes with *Q* value < 0.01 and |logFC| > 2 were chosen as differentially expressed genes (DEGs) and the results were graphed in a volcano plot. Protein interaction analysis of DEGs was performed using the Search Tool for the Retrieval of Interacting Genes/Proteins database. Cytoscape was used to screen the core modules and genes. KEGG pathway annotation was visualized by using the BGI enrichment analysis tool.

### Screening for Potential Transcription Factors That Regulate MARCH3

JASPA website (http://jaspar.genereg.net/) was used to predict the transcription factors that could bind to the promoter region of MARCH3. Taking the intersection of DEGs and predicted transcription factors, the optimal potential target genes were obtained. The gene list of DEGs and predicted transcription factors are listed in Supporting Information‐gene list.

### Coimmunoprecipitation (co‐IP) and Ubiquitination Analyses

HepG2 cells were collected and lysed using a cell lysis buffer for western blotting and IP (1 mL, Beyotime, China) supplemented with a protease inhibitor cocktail (Beyotime, China). Protein G Sepharose (30 µL, GE Healthcare, UK) and normal IgG antibodies (1 µg, Proteintech, China) were used to block the lysates (4 °C, 1 h). Next, the lysate was incubated overnight with HA (1 µg) or normal IgG (1 µg) antibodies at 4 °C and then incubated with protein G Sepharose (30 µL) at 4 °C for 3 h to conduct the co‐IP assay. The immunoprecipitate was denatured at 100 °C for 5 min in the sample buffer. For ubiquitination analysis, HepG2 cells were treated with 20 × 10^−6^ m MG132 (MCE, China) for 3 h. The cells were then collected, and a co‐IP assay was performed. Finally, ubiquitinated proteins were detected using a UB antibody (ABclonal, China).

### Histological Analysis

Hematoxylin and eosin (H&E) and Oil red O staining were performed to visualize lipid accumulation in the liver as previously reported.^[^ ^33^ ^]^ Prussian blue staining was performed using a kit (Solarbio, China), according to the manufacturer's instructions. Antigen retrieval was performed using 0.1 m sodium citrate buffer. Sections were subsequently blocked using an immunohistochemistry (IHC) kit (Maixin, China). Subsequently, the sections were incubated overnight with primary antibodies at 4 °C. The next day, the slides were incubated with a biotin‐labeled secondary antibodies for 1 h at room temperature, after which the sections were counterstained with hematoxylin. Finally, images were acquired using a polarized light microscope (Olympus, Japan). The primary antibodies used were Caspase3 cle (CST, USA), MLKL (Affinity, China), ATG5 (CST, USA), and GPX4 (ABclonal, China).

### Transmission Electron Microscopy

The cells were fixed with 1% osmic acid (0.1 m) in phosphate‐buffered saline (PBS, pH 7.4). The cells were subsequently stained with uranyl acetate and lead citrate, embedded in Epon, sliced into ultrathin sections, and visualized under a transmission electron microscope (FEI, USA).

### Immunofluorescence

The cells were fixed with 4% paraformaldehyde for 30 min, incubated with 0.5% Triton X‐100 solution for 20 min, and treated with 5% BSA blocking solution for 1 h at room temperature. The cells were then incubated overnight with anti‐4HNE (Abcam, China) primary antibodies at 4 °C. The following day, cells were incubated with goat anti‐rabbit IgG (H+L) (Highly Cross‐Adsorbed Secondary Antibody; Thermo Fisher Scientific, USA) for 1 h at room temperature. Cells were visualized using an inverted fluorescence microscope (Olympus, Japan).

### Statistical Analysis

GraphPad Prism 9.12 (GraphPad, USA) was used for statistical analysis. All the data are presented as the means ± standard deviation (mean±SD) of at least three independent experiments. Statistical significance was determined by Student's *t*‐test for comparisons between two groups and one‐way ANOVA for comparisons among more than two groups. Differences were considered to be statistically significant at *P* < 0.05 (^*^ *p* < 0.05, ^**^ *p* < 0.01, ^***^ *p* < 0.001).

### Ethics Approval and Consent to Participate

This study was approved by the Institutional Ethics Board of Zhongnan Hospital of Wuhan University (No.2019021). All patients were informed about the procedure and provided informed consent. The animal studies were approved by the Zhongnan Hospital Animal Ethics committee(No.WP20220036).

## Conflict of Interest

The authors declare no conflict of interest.

## Author Contributions

W.C., S.W., X.M., and Z.L. contributed equally to this work. W.C.: conceptualization, methodology, formal analysis, investigation, data curation, and writing‐original draft. S.W.: conceptualization, methodology, formal analysis, investigation, data curation, and writing‐original draft. X.M.: methodology, formal analysis, investigation, data curation, and writing‐original draft. Z.L.: methodology, software, formal analysis, investigation, data curation, and writing‐original draft. D.P. and X.Y.: resources and data curation. M.Y.: data curation and visualization. Y.Y. and X.C.: writing–review & editing, funding acquisition, and supervision.

## Supporting information

Supporting Information

Supporting Information

## Data Availability

The data that support the findings of this study are available from the corresponding author upon reasonable request.
