# Supplementary material for: IL6 Derived from Macrophages under Intermittent Hypoxia Exacerbates NAFLD by Promoting Ferroptosis via MARCH3‐Led Ubiquitylation of GPX4
Source: Adv Sci (Weinh). 2024 Sep 4;11(41):2402241. doi: 10.1002/advs.202402241 (PMC11538716; doi:10.1002/advs.202402241)
Supplement: Supplementary file 1 — Supporting Information [file ADVS-11-2402241-s002.docx]

Supporting Information

IL6 Derived from Macrophages under Intermittent Hypoxia Exacerbates NAFLD by Promoting Ferroptosis via MARCH3-led Ubiquitylation of GPX4

Weisong Cai, Sa Wu, Xiaoping Ming, Zhen Li, Dingyu Pan, Xiuping Yang, Minlan Yang, Yufeng Yuan，Xiong Chen

**Supplementary Figure 1.The lowest arterial oxygen saturation data for both patients and mice.**


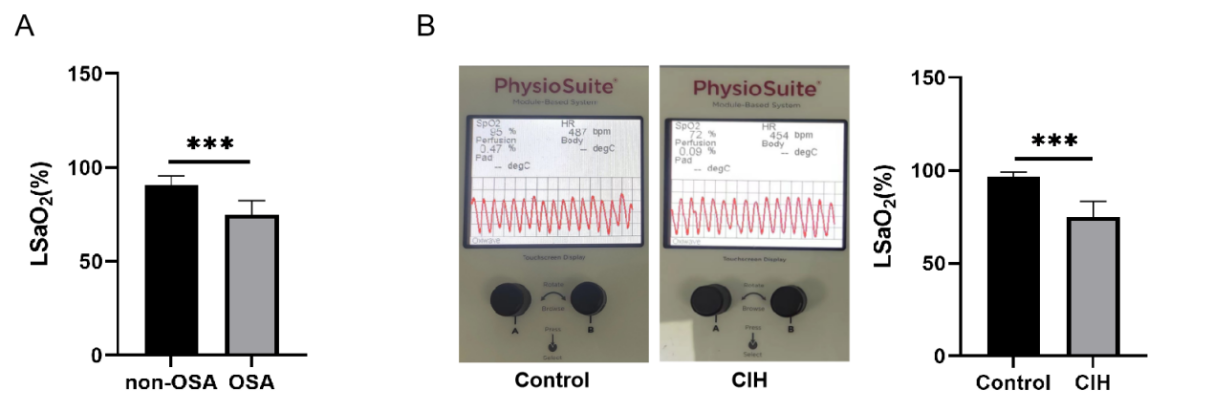


Supplementary Figure 1.The lowest arterial oxygen saturation data for both patients (non-OSA=10, OSA=10)(A) and mice (n=5)(B). (*** *P*< 0.001).

**Supplementary Figure 2. Transcriptional consequences of hypoxia signaling for** **patients with OSA.**

**
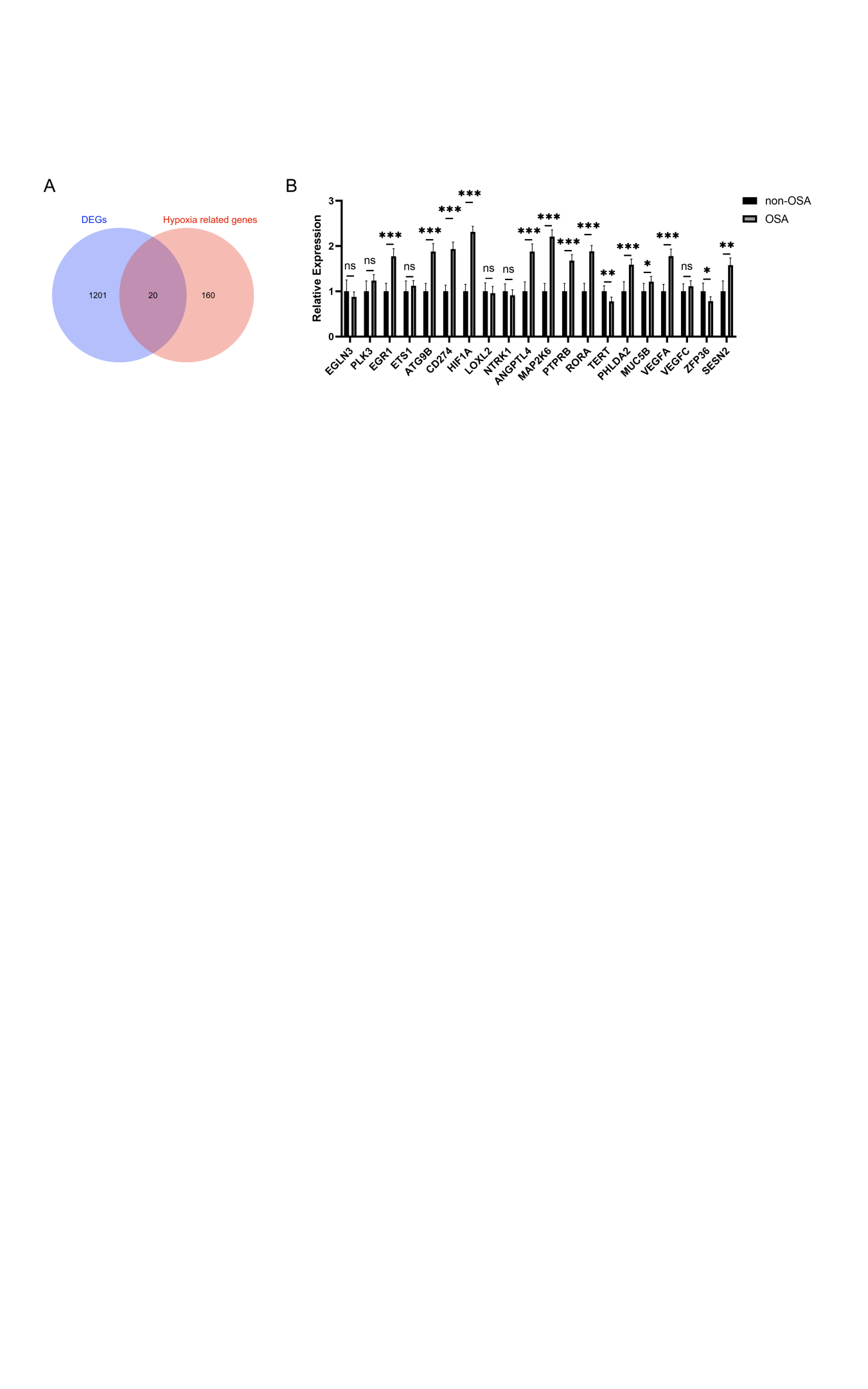
**

Supplementary Figure 2. Transcriptional consequences of hypoxia signaling for patients with OSA. A. Hypoxia related genes was downloaded from Genecards database, and intersection of DEGs and hypoxia related genes was shown using a Venn diagram. B. Hypoxia related genes mRNA levels of patients’ livers were evaluated by q-PCR. (non-OSA=10, OSA=10)(**P* < 0.05; ***P*< 0.01; ****P*< 0.001; ns, not significant).

**Supplementary Figure 3.** **HIF1A inhibitor alleviated MARCH3-led Ubiquitylation of GPX4.**

**
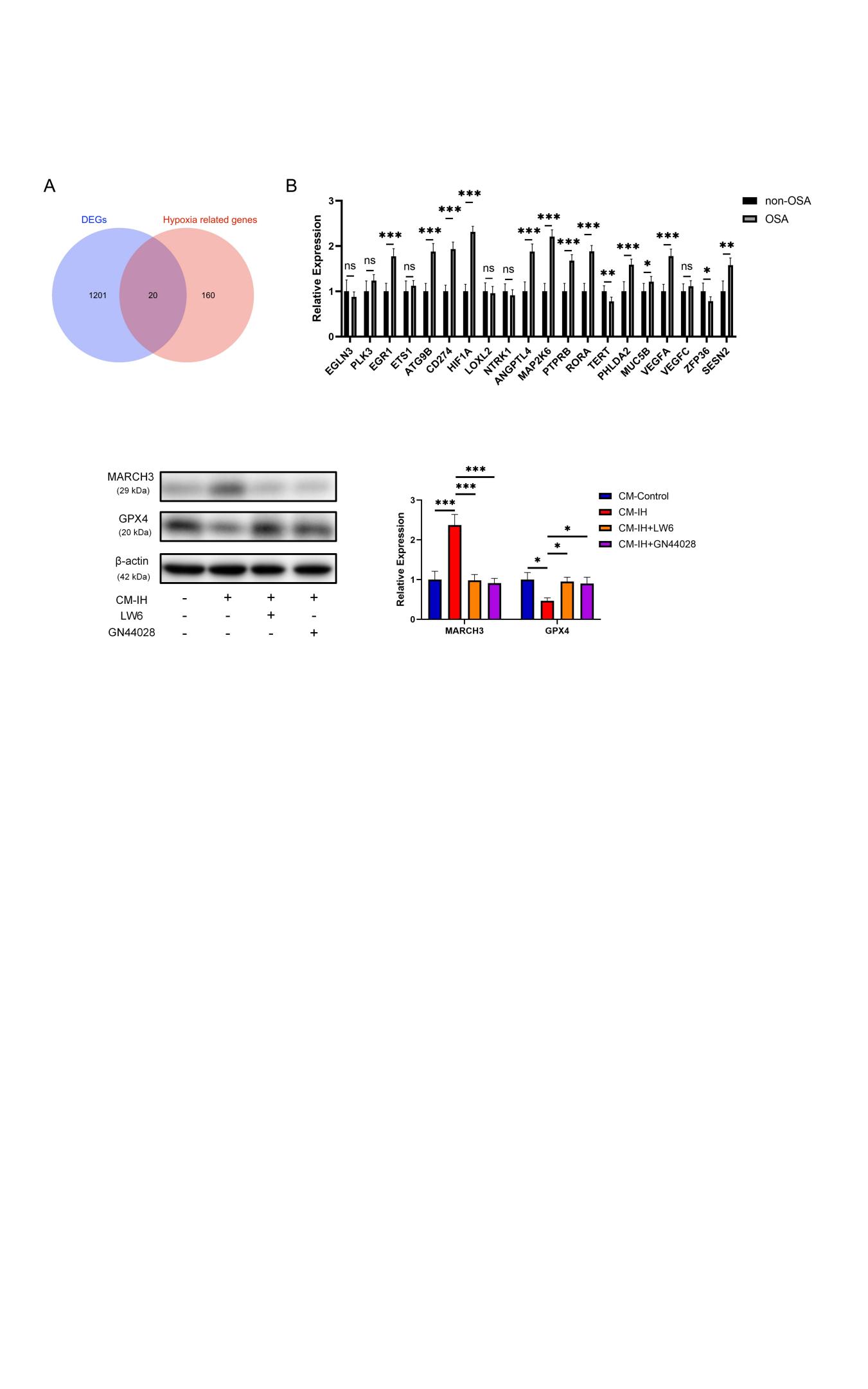
**

Supplementary Figure 3. HIF1A inhibitor alleviated MARCH3-led Ubiquitylation of GPX4. The protein levels of MARCH3 and GPX4 in the HepG2 cells treated with HIF1A inhibitor LW6 (20 μm, 24 h) or GN44028 (40 μm, 24 h) under CM-IH (24 h) condition. (*P < 0.05; *** P< 0.001).

**Supplementary Figure 4. DFO alleviated CIH-induced liver function injury in HFD-fed mice.**

**
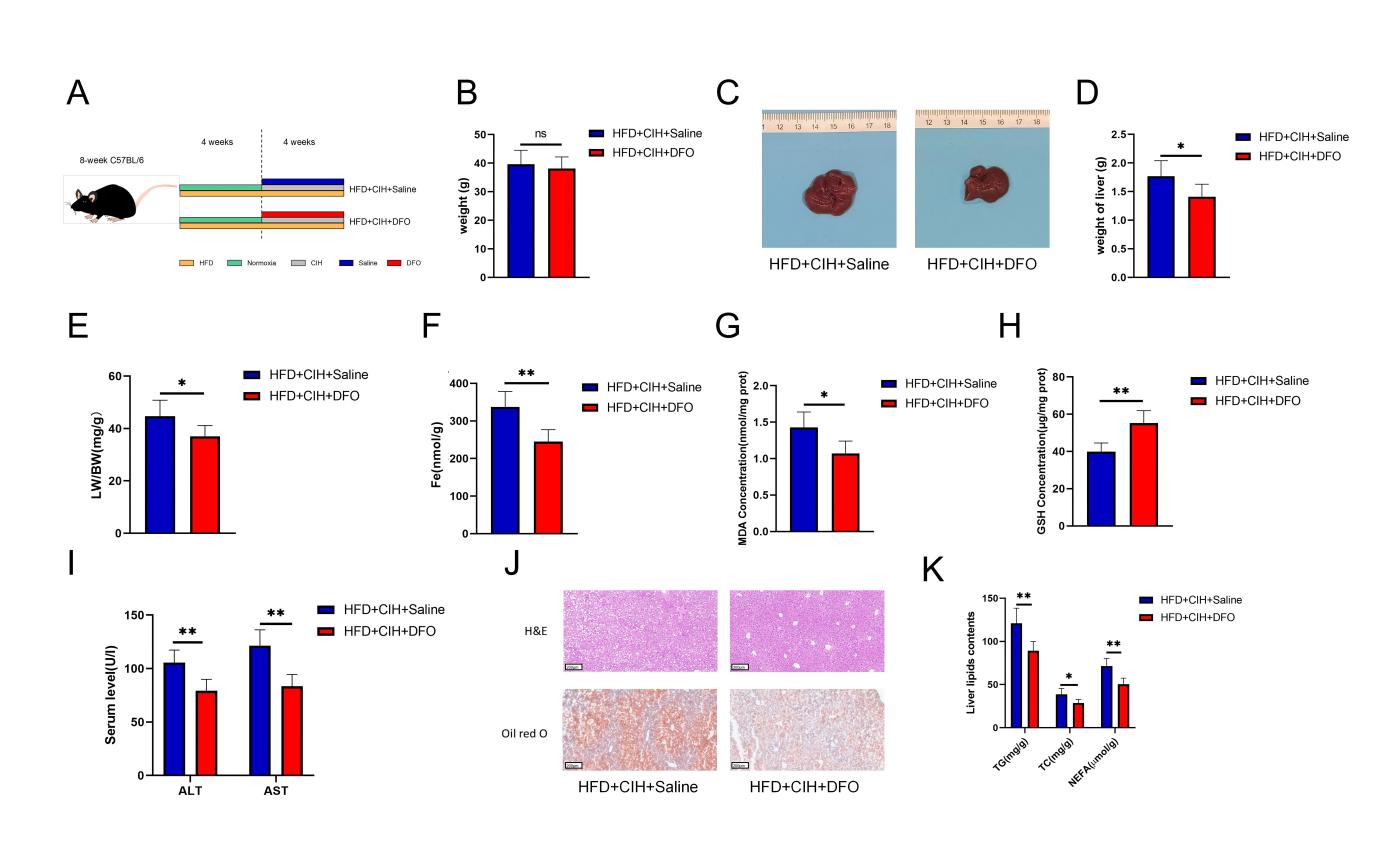
**

Supplementary Figure 4. DFO alleviated CIH-induced liver function injury in HFD-fed mice. A. Interventions for the different groups of mice (n=5, 200 mg/kg DFO). B-E. Weight (B), representative images (C), liver weight (D) and LW/BW ratio (E) of the mice in the HFD+CIH+saline group and the HFD+CIH+DFO group. F-H. Ferroptosis indices showed the concentrations of Fe (F), MDA (G), and GSH (H) in mouse liver tissues from the two groups. I. The mouse serum levels of ALT and AST in the two groups. J. H&E staining and Oil red O staining of mouse liver tissues (scale bar, 100 μm). K. Mouse serum TG, TC and NEFA levels.  (**P* < 0.05; ** *P*< 0.01).

**Supplementary Figure 5.** **CIH significantly increased the IL6 level of mice liver.**

**
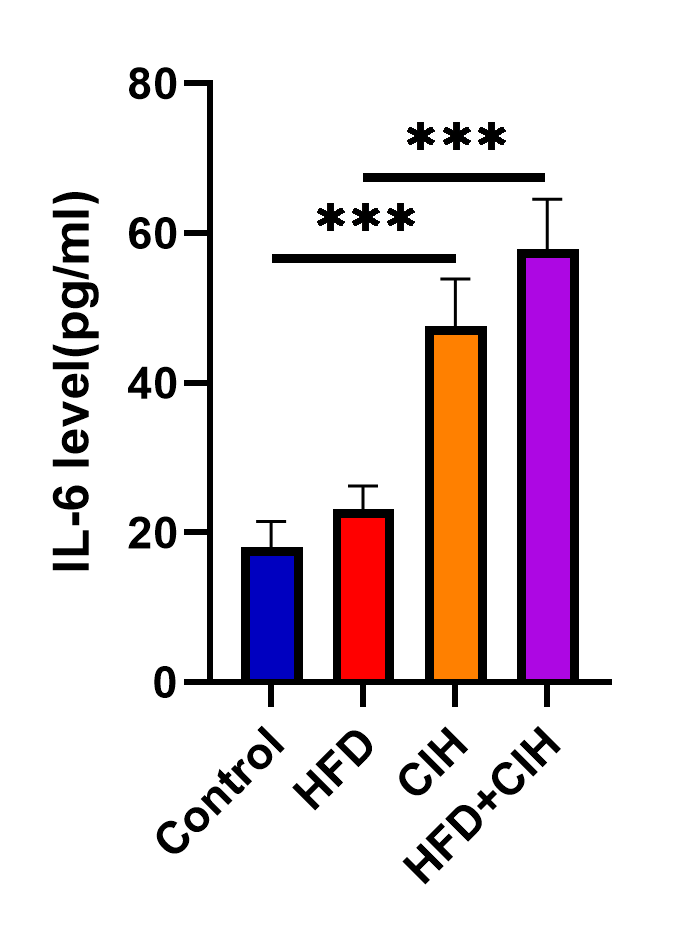
**

Supplementary Figure 5. CIH significantly increased the IL6 level of mice liver. IL6 concentration in the mice liver was determined by ELISA. (n=5 per group)(*** *P*< 0.001).

**Supplementary Figure 6. The transcription of MARCH3** **in HepG2 cells under IH or chronic hypoxia.**

**
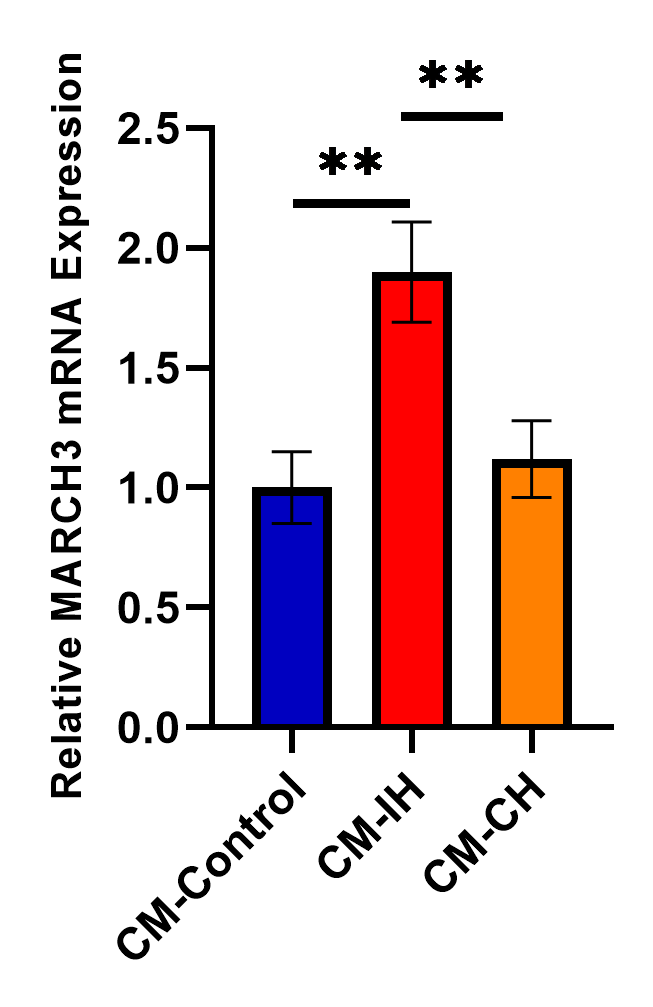
**

Supplementary Figure 6.The transcription of MARCH3 in HepG2 cells under IH or chronic hypoxia. CM-Control: supernatants derived from macrophages under normoxia; CM-IH:supernatants derived from macrophages under intermittent hypoxia. CM-CH: supernatants derived from macrophages under chornic hypoxia. (** *P*< 0.01).

**Supplementary Figure 7.** **MARCH3 expression increased in the liver of patients with OSA.**

**
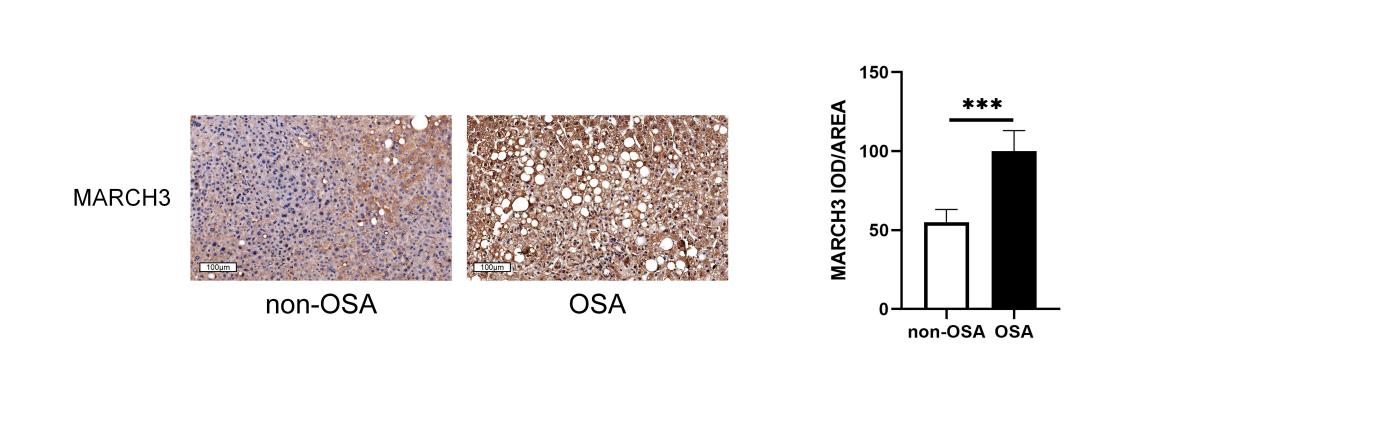
**

Supplementary Figure 7. MARCH3 expression increased in the liver of patients with OSA. IHC results of MARCH3 in liver tissues of patients (non-OSA=10, OSA=10)(*** *P*< 0.001).

**Supplementary Figure 8. Western** **blotting showed MARCH3 knockdown efficiency of three siRNAs.**


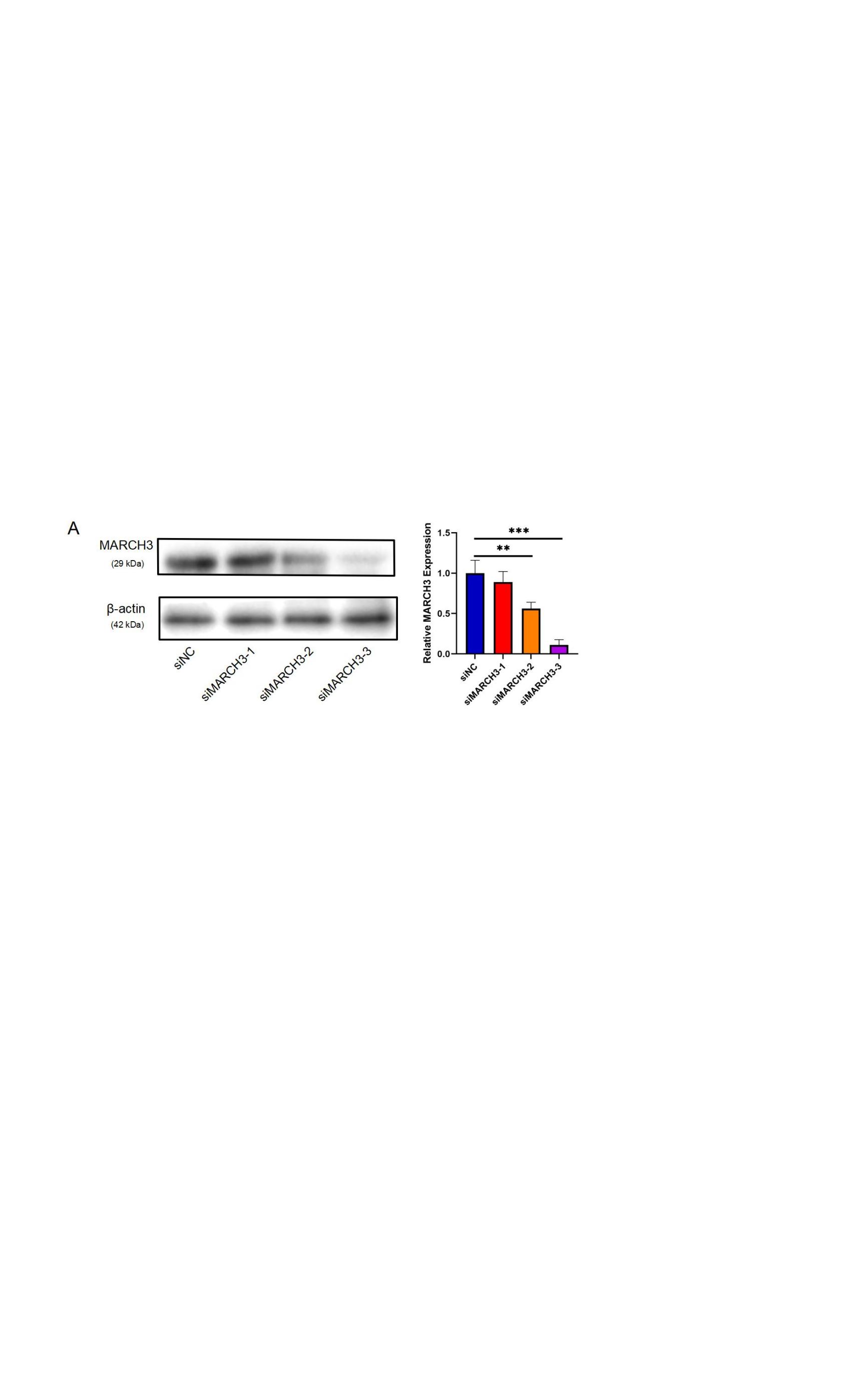


Supplementary Figure 8. Western blotting showed MARCH3 knockdown efficiency of three siRNAs. (** *P*< 0.01; *** *P*< 0.001).

**Supplementary Figure 9.** **Knockdown of MARCH3 alleviated IL-6 induced ferroptosis in HepG2 and LO_2_ cells under IH.**


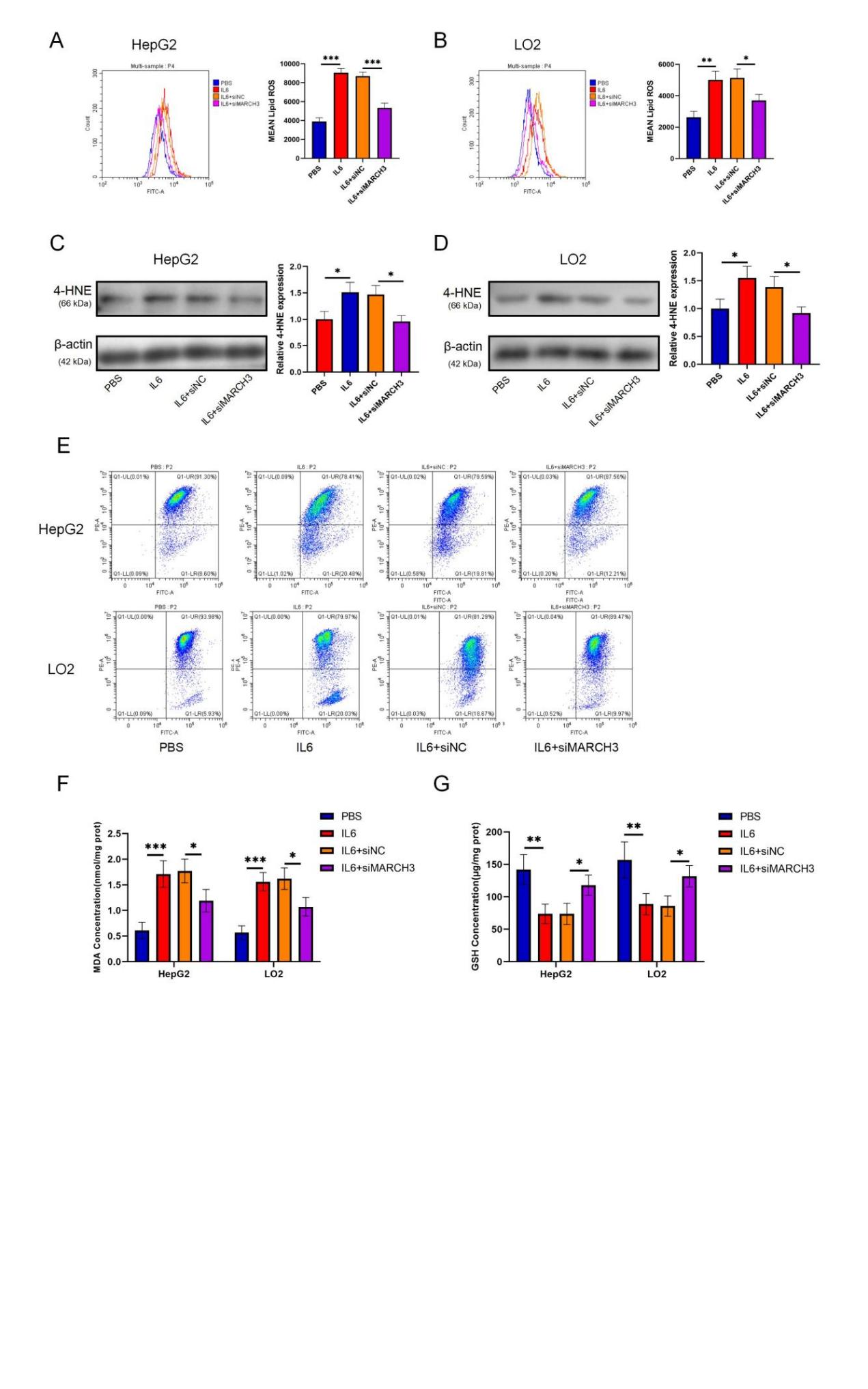


Supplementary Figure 9. Knockdown of MARCH3 alleviated IL-6 induced ferroptosis in HepG2 and LO2 cells under IH. A-B. The level of lipid ROS via flow cytometry. C-D. Western blotting showed 4-HNE level. E. Mitochondrial membrane potential was assessed by JC-1 staining, which revealed mitochondria with high (increased PE) or low (increased FITC) membrane potential. F. MDA level. G.GSH content.  (**P* < 0.05; ** *P*< 0.01; *** *P*< 0.001).

**Supplementary Figure 10. Levels of RUNX1, MARCH3, and GPX4 mRNA were detected in *vivo* and in *vitro*.**


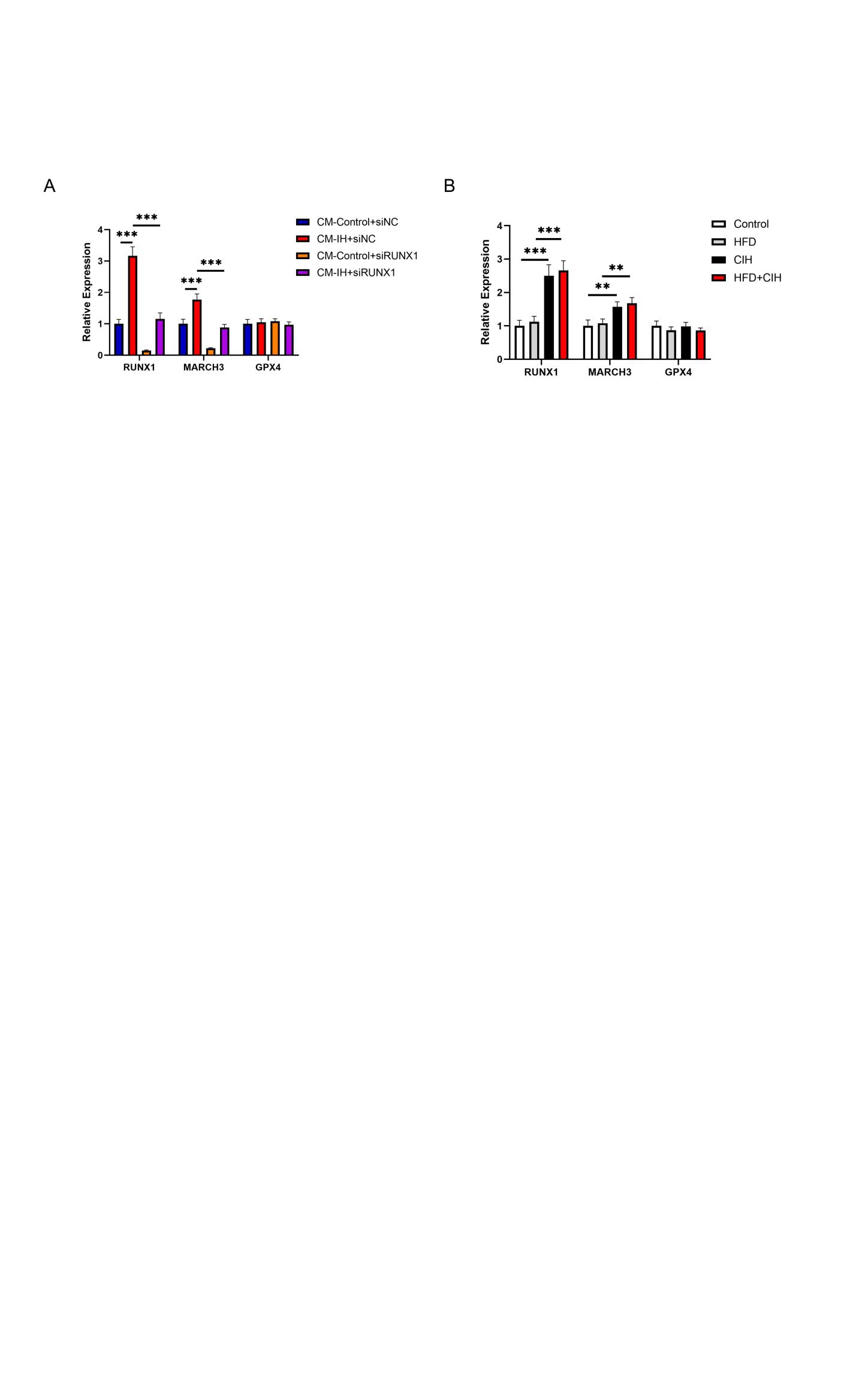


Supplementary Figure 10. Levels of RUNX1, MARCH3, and GPX4 mRNA were detected in *vivo* and in *vitro*. A. Levels of RUNX1, MARCH3, and GPX4 in HepG2 cells treated with CM-IH and siRUNX1. B. Levels of RUNX1, MARCH3, and GPX4 in Control, HFD, CIH, and HFD+CIH mice after eight-weeks intervention (n=5 per group) (** *P*< 0.01; *** *P*< 0.001).

**Supplementary Figure 11. IHC results of MARCH3 and GPX4 in mice liver tissues.**


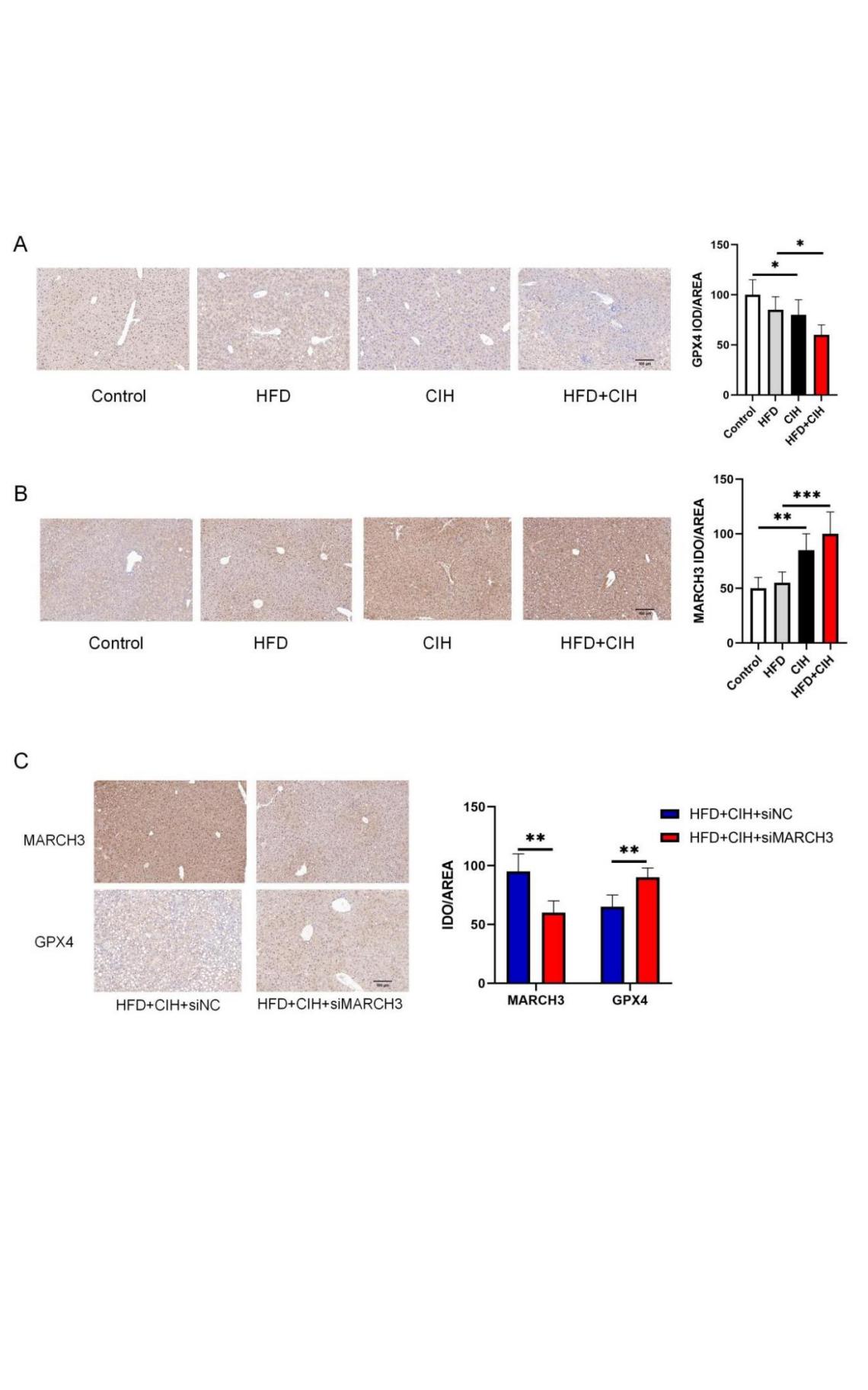


Supplementary Figure 11. IHC results of MARCH3 and GPX4 in mice liver tissues. A-B. GPX4 and MARCH3 levels in mice liver tissues from control, HFD, CIH and HFD+CIH mice after eight-weeks intervention (n=5 in each group). C. GPX4 and MARCH3 levels in mice liver tissues from HFD+CIH+siNC group and HFD+CIH+siMARCH3 group. (**P* < 0.05; ** *P*< 0.01; *** *P*< 0.001).

**Table S1-Primers**

| Gene | Sequence |
| --- | --- |
| mice HMGCR for | ATCATGTGCTGCTTCGGCTGCAT |
| mice HMGCR rev | AAATTGGACGACCCTCACGGCT |
| mice CYP7A1 for | TCAAAGAGCGCTGTCTGGGTCA |
| mice CYP7A1 rev | TTTCCCGGGCTTTATGTGCGGT |
| mice ABCG1 for | TGAACCCGTTTCTTTGGCACCG |
| mice ABCG1 rev | AGTCCCGCATGATGCTGAGGAA |
| mice FATP1 for | TGCACAGCAGGTACTACCGCAT |
| mice FATP1 rev | TGCGCAGTACCACCGTCAAC |
| mice FABP-1 for | TGGTCCGCAATGAGTTCACCCT |
| mice FABP-1 rev | CCAGCTTGACGACTGCCTTGACTT |
| mice CD36 for | TGGGTTTTGCACATCAAAGA |
| mice CD36 rev | GATGGACCTGCAAATGTCAGA |
| mice SREBP-1c for | CACTTCTGGAGACATCGCAAAC |
| mice SREBP-1c rev | ATGGTAGACAACAGCCGCATC |
| mice FAS for | CTGCGGAAACTTCAGGAAATG |
| mice FAS rev | GGTTCGGAATGCTATCCAGG |
| mice ACCα for | GGCCAGTGCTATGCTGAGAT |
| mice ACCα rev | AGGGTCAAGTGCTGCTCCA |
| mice PPARγ for | ATTCTGGCCCACCAACTTCGG |
| mice PPARγ rev | TGGAAGCCTGATGCTTTATCCCCA |
| mice PDK4 for | TTCACACCTTCACCACATGC |
| mice PDK4 rev | AAAGGGCGGTTTTCTTGATG |
| mice PPAR-α for | TATTCGGCTGAAGCTGGTGTAC |
| mice PPAR-α rev | CTGGCATTTGTTCCGGTTCT |
| mice CPT-1α for | AGGACCCTGAGGCATCTATT |
| mice CPT-1α rev | ATGACCTCCTGGCATTCTCC |
| mice ACOX-1 for | CGGAAGATACATAAAGGAGACC |
| mice ACOX-1 rev | AAGTAGGACACCATACCACCC |
| mice MCAD for | TGGCGTATGGGTGTACAGGG |
| mice MCAD rev | CCAAATACTTCTTTTTTTGTTGATCA |
| mice UCP2 for | GCTGGTGGTGGTCGGAGATA |
| mice UCP2 rev | ACTGGCCCAAGGCAGAGTT |
| human EGLN3 for | CTGGGCAAATACTACGTCAAGG |
| human EGLN3 rev | GACCATCACCGTTGGGGTT |
| human PLK3 for | AGCGCCTACGCTGTCAAAG |
| human PLK3 rev | CTCAAAGTGGTGCGAAAAACG |
| human EGR1 for | GGTCAGTGGCCTAGTGAGC |
| human EGR1 rev | GTGCCGCTGAGTAAATGGGA |
| human ETS1 for | GATAGTTGTGATCGCCTCACC |
| human ETS1 rev | GTCCTCTGAGTCGAAGCTGTC |
| human ATG9B for | CCCCTCATACAAGAAGCTCCC |
| human ATG9B rev | TGCAGGTTGAGCCTGTGTTG |
| human CD274 for | TGGCATTTGCTGAACGCATTT |
| human CD274 rev | TGCAGCCAGGTCTAATTGTTTT |
| human HIF1A for | GAACGTCGAAAAGAAAAGTCTCG |
| human HIF1A rev | CCTTATCAAGATGCGAACTCACA |
| human LOXL2 for | CACTATGACCTGCTGAACCTCAATGG |
| human LOXL2 rev | TGGCACACTCGTAATTCTTCTGGATG |
| human NTRK1 for | AACCTCACCATCGTGAAGAGT |
| human NTRK1 rev | TGAAGGAGAGATTCAGGCGAC |
| human ANGPTL4 for | GTCCACCGACCTCCCGTTA |
| human ANGPTL4 rev | CCTCATGGTCTAGGTGCTTGT |
| human MAP2K6 for | GAAGCATTTGAACAACCTCAGAC |
| human MAP2K6 rev | CCTGGCTATTTACTGTGGCTC |
| human PTPRB for | GGGCTCACCCTGTAACTTTAGC |
| human PTPRB rev | TCTATCCGAAAGGTAGGGCAC |
| human RORA for | ACTCCTGTCCTCGTCAGAAGA |
| human RORA rev | CATCCCTACGGCAAGGCATTT |
| human TERT for | AAATGCGGCCCCTGTTTCT |
| human TERT rev | CAGTGCGTCTTGAGGAGCA |
| human PHLDA2 for | TCACCACCGACCACAAGGAGATC |
| human PHLDA2 rev | TTCCTGGCGGCTGCGAAAGT |
| human MUC5B for | GCCTACGAGGACTTCAACGTC |
| human MUC5B rev | CCTTGATGACAACACGGGTGA |
| human VEGFA for | AGGGCAGAATCATCACGAAGT |
| human VEGFA rev | AGGGTCTCGATTGGATGGCA |
| human VEGFC for | GAGGAGCAGTTACGGTCTGTG |
| human VEGFC rev | TCCTTTCCTTAGCTGACACTTGT |
| human ZFP36 for | GACTGAGCTATGTCGGACCTT |
| human ZFP36 rev | GAGTTCCGTCTTGTATTTGGGG |
| human SESN2 for | AAGGACTACCTGCGGTTCG |
| human SESN2 rev | CGCCCAGAGGACATCAGTG |
