# Supplementary material for: IL6 Derived from Macrophages under Intermittent Hypoxia Exacerbates NAFLD by Promoting Ferroptosis via MARCH3‐Led Ubiquitylation of GPX4
Source: Adv Sci (Weinh). 2024 Sep 4;11(41):2402241. doi: 10.1002/advs.202402241 (PMC11538716; doi:10.1002/advs.202402241)

细胞遗传质量鉴定检测

Cell Line Authentication Service

STR 基因型检测报告

样 品 信 息

样品编号：

客户样本编号	公司编号
8	20211012-01

样品数量：1

样品性状：细胞系

检测项目：STR

送检单位：宁波明舟生物科技有限公司

检测方法：用 Axygen 的基因组抽提试剂盒提取 DNA，采用 20- STR 扩增方案扩增，在 ABI 3730XL 型遗传分析仪上对 STR 位点和性别基因 Amelogenin 进行检测。

检 测 结 果

(一)　检验基本情况

	多等位基因	匹配细胞系	细胞库	EV 值	匹配说明
20211012-01	有	HEP-G2	ATCC	0.97	基本匹配

样本基因型检验结果

- 多等位基因指三等位及以上基因现象。

- 本次检测各细胞分型结果良好。

(二)　各样本描述

- 20211012-01：该株细胞 DNA 分型在细胞系检索中找到**基本匹配**的细胞系，ATCC 数据库显示细胞名为 **HEP-G2**，细胞号对应 **H**B-**80**65。本次检测在该细胞系中**发现多等位基因**。

备注：待测细胞系与收录于 ATCC, DSMZ, JCRB 和 RIKEN 数据库的细胞系 STR 数据进行比对，未收录于以上细胞库的细胞系将无法匹配。

（三）样本分型结果

细胞 20211012-01 的 STR 位点和 Amelogenin 位点的基因分型结果						
Loci	送检细胞 STR 信息			细胞库细胞 STR 信息		
	送检细胞名：8			细胞库细胞名：HEP-G2		
	Allele1	Allele2	Allele3	Allele1	Allele2	Allele3
D5S818	11	12	13	11	12	
D13S317	9	13		9	13	

D7S820	10	10		10	10	
D16S539	12	13		12	13	
VWA	17	17		17	17	
TH01	9	9		9	9	
AMEL	X	Y		X	Y	
TPOX	8	9		8	9	
CSF1PO	10	11		10	11	
D12S391	21	25				
FGA	22	25				
D2S1338	19	20				
D21S11	29	30	31			
D18S51	13	14				
D8S1179	15	16				
D3S1358	15	16				
D6S1043	13	13				
PENTAE	15	20				
D19S433	15.2	15.2				
PENTAD	9	13				

其他说明

(一) 分型方案及位点分布

	方案 1	方案 2	方案 3	方案 4
1	TH01	TPOX	D3S1358	AMEL
2	D12S391	VWA	D13S317	D5S818
3	D7S820	D8S1179	D6S1043	D2S1338
4	CSF1PO	PENTAD	D16S539	D21S11
5	FGA		D19S433	D18S51
6	PENTAE			

实验方案及位点

(二) STR 数据库比对

本公司采用 DSMZ tools 进行细胞系比对，其中包含来自于 ATCC, DSMZ, JCRB 和 RIKEN 数据库的 2455 个细胞系 STR 数据。如果待检测细胞未收录于以上细胞库或这是自行建立的新细胞系将无法进行比对，用户需根据细胞分型结果自行与其他数据库进行比对。

签发日期：2021年 10 月 25 日

Applied Biosystems
GeneMapper 4.0

Cell Line Authentication 1012 16 18

Sample File	Sample Name	Panel	OS	SQ
02_D02.fsa	8	STR Profile 1-human		

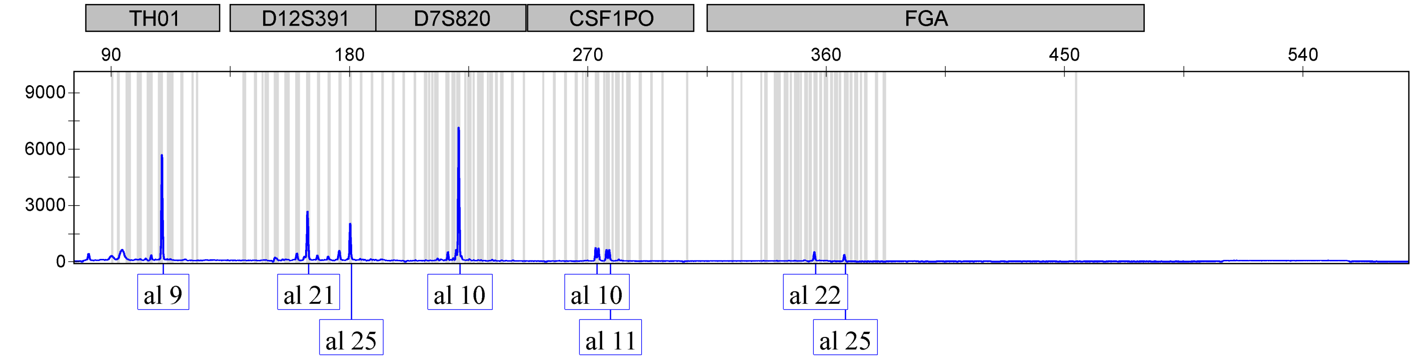

Sample File	Sample Name	Panel	OS	SQ
02_D02.fsa	8	STR Profile 1-human		

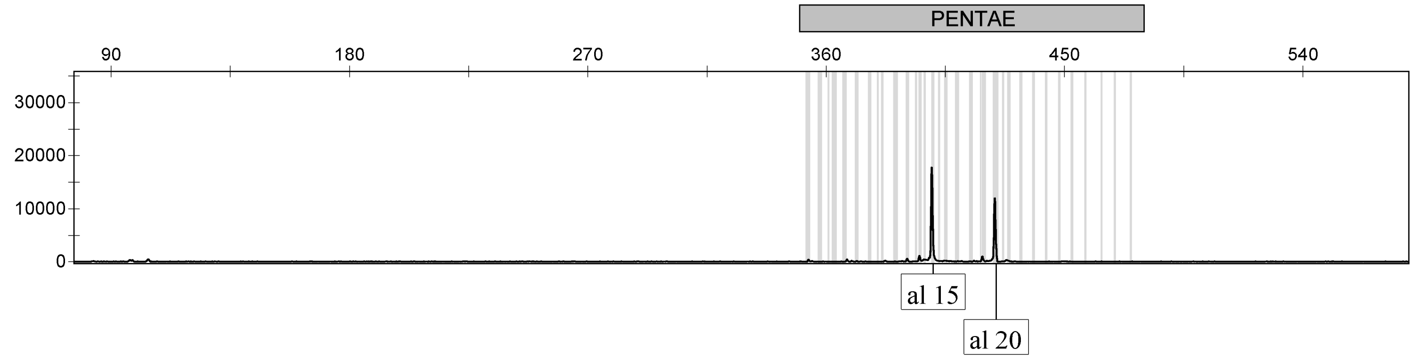

Sample File	Sample Name	Panel	OS	SQ
11_D11.fsa	8	STR Profile 2-human		

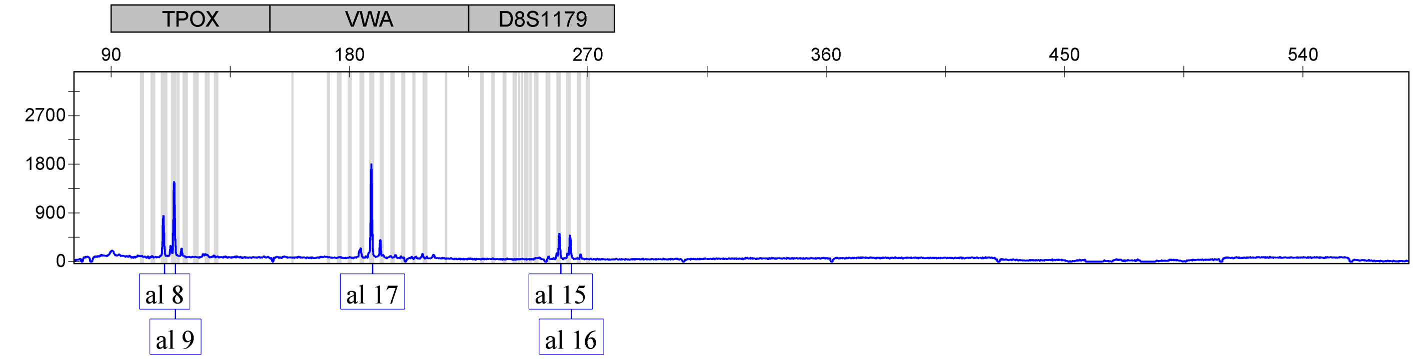

Applied Biosystems
GeneMapper 4.0

Cell Line Authentication 1012 16 18

Sample File	Sample Name	Panel	OS	SQ
11_D11.fsa	8	STR Profile 2-human		

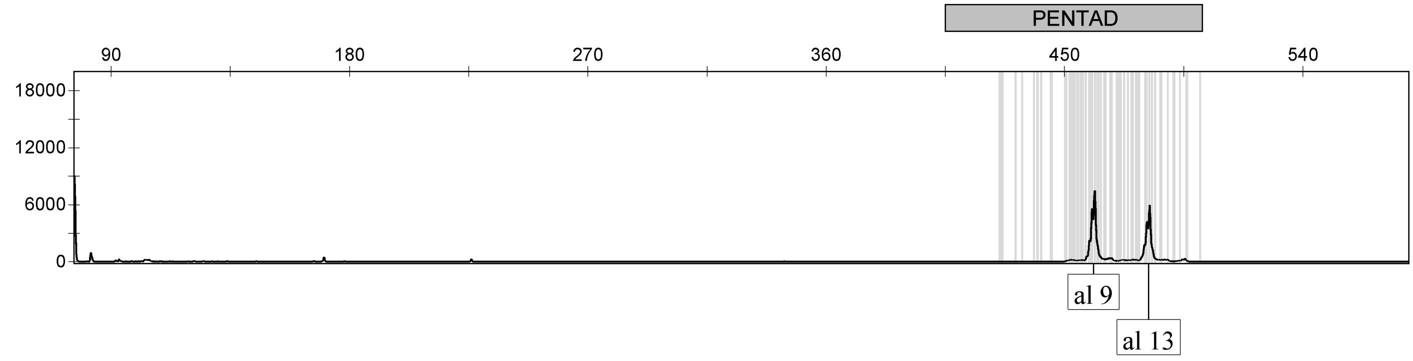

Sample File	Sample Name	Panel	OS	SQ
08_D08.fsa	8	STR Profile 3-human		

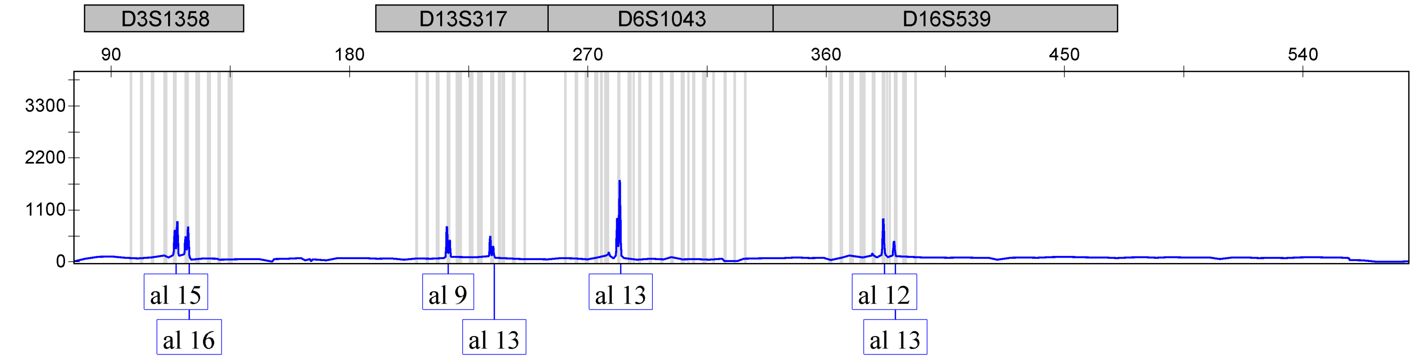

Sample File	Sample Name	Panel	OS	SQ
08_D08.fsa	8	STR Profile 3-human		

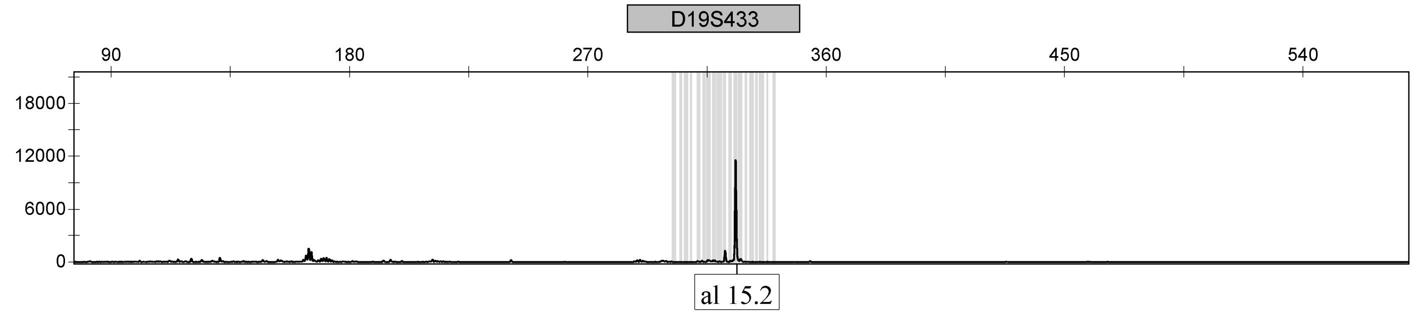

GeneMapper 4.0

Cell Line Authentication 1012 16 18

Sample File	Sample Name	Panel	OS	SQ
05_D05.fsa	8	STR Profile 4-human		

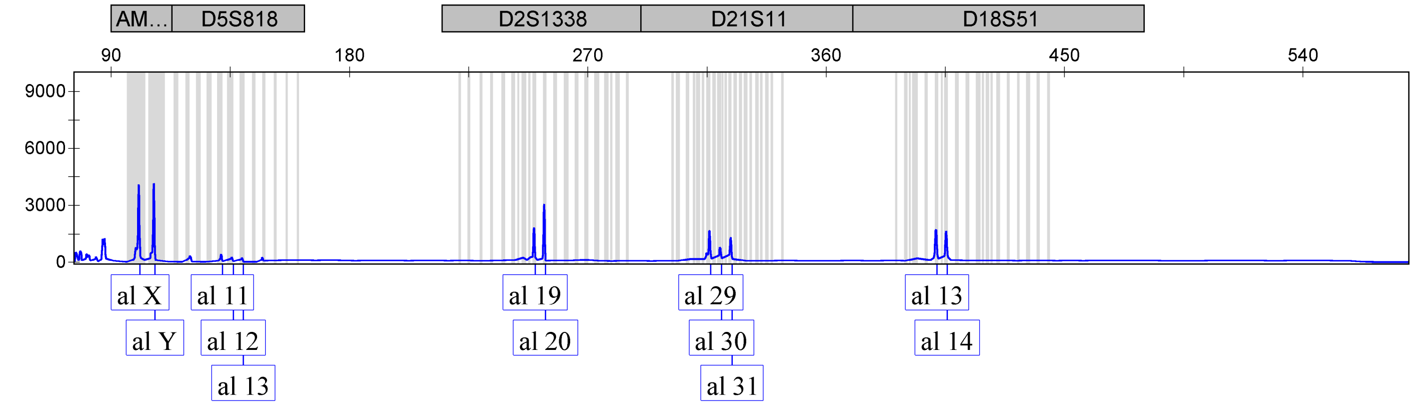

05_D05.fsa	8	STR Profile 4-human		

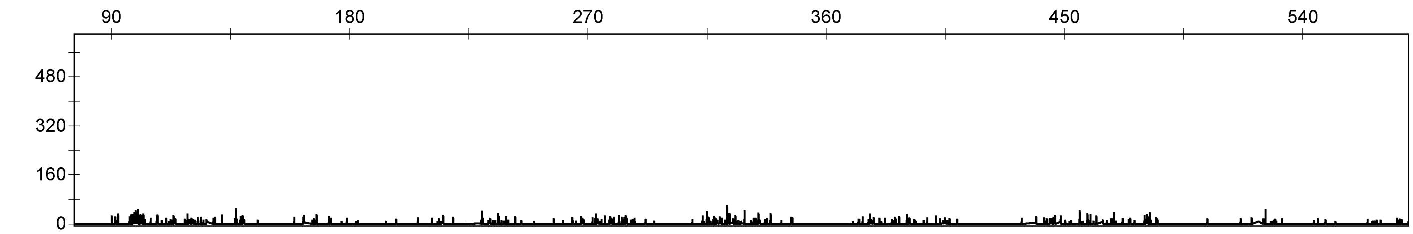

Supplement: Supplementary file 2 — Supporting Information [file ADVS-11-2402241-s001.zip › STR genotype test report-HepG2.pdf]
